# Revision of the freshwater genus *Atyaephyra* (Crustacea, Decapoda, Atyidae) based on morphological and molecular data

**DOI:** 10.3897/zookeys.229.3919

**Published:** 2012-10-19

**Authors:** Magdalini Christodoulou, Aglaia Antoniou, Antonios Magoulas, Athanasios Koukouras

**Affiliations:** 1 Department of Zoology, School of Biology, Aristotle University of Thessaloniki, 54124 Thessaloniki, Macedonia, Greece; 2 Institute of Marine Biology and Genetics, Hellenic Centre for Marine Research, Gournes Pediados, 71003, Heraklion, Crete, Greece

**Keywords:** Atyidae, *Atyaephyra*, new species, cryptic species, COI, freshwater shrimp, molecular data, morphology, taxonomy

## Abstract

*Atyaephyra* de Brito Capello, 1867 was described from the Mediterranean region almost 200 years ago. Since then, the genus has been recorded from various freshwater habitats in Europe, North Africa and the Middle East. Despite its long history, the taxonomic status of *Atyaephyra* species remains confusing and uncertain. Consequently numerous specimens from the known range of *Atyaephyra* were analysed using morphological characters and mitochondrial COI sequences in an attempt to clarify the taxonomy of this genus. The present study recognises seven *Atyaephyra* species, more than twice as many as previously recorded (three), four of which are considered as new. The new species are described, additional information to the original descriptions are provided for the remaining three taxa, while neotypes of *Atyaephyra desmarestii* Millet, 1831 and *Atyaephyra stankoi* Karaman, 1972 are designated to stabilize their taxonomy. Non-overlapping distinguishing morphological characters are used to discriminate the examined material into five species, e.g., *Atyaephyra desmarestii*, *Atyaephyra stankoi*, *Atyaephyra orientalis* Bouvier, 1913, *Atyaephyra thyamisensis* **sp. n.**, *Atyaephyra strymonensis* **sp. n.** In addition, the genetic analysis supports the existence of multiple phylogenetic clades in the broader Mediterranean area and distinguishes two new cryptic species, namely *Atyaephyra tuerkayi* **sp. n.** and *Atyaephyra acheronensis* **sp. n.** The geographic distribution of these species is confirmed and their phylogenetic relationships are described.

## Introduction

Atyidae is one of the most diverse shrimp families comprising at least 469 valid species (De Grave and Fransen 2011) being found in freshwater habitats world-wide with the exception of Antarctica. However, this high number of species is probably an underestimate of the family’s species richness. The latter becomes evident given the current indication of numerous, yet undescribed species, many of which being characterized as cryptic (Cook et al. 2006, Page and Hughes 2007, Page et al. 2008, Cook et al. 2008) and pending further research to be confirmed or not as such. Currently, 43 atyid genera (De Grave and Fransen 2011, Richard et al. 2012) have been established, five of which (*Atyaephyra* de Brito Capello, 1867, *Dugastella* Bouvier, 1912, *Gallocaris* Sket and Zakšek, 2009, *Typhlatya* Creaser, 1936, *Troglocaris* Dormitzer, 1853) are found in the broader Mediterranean region.

*Atyaephyra* is the most widespread atyid taxon in the Mediterranean region with its native range spanning from the Middle East to North Africa, a large part of Southern Europe and to some Mediterranean islands (Corsica, Sardinia, Sicily) (d’ Udekem d’ Acoz 1999). Furthermore, it has been introduced into North and Central Europe through river canals opened in France (e.g. Dhur and Massard 1995, Moog et al. 1999, Grabowski et al. 2005, Straka and Špaček 2009).

*Atyaephyra* was first reported in the Mediterranean region almost 200 years ago (Rafinesque 1814) and like most of old taxa has a very confused taxonomic history. The oldest species of *Atyaephyra* (*Atyaephyra desmarestii*) and only one until recently, was first described by Rafinesque (1814) as *Symethus fluviatilis*, based on material most likely collected from Simeto River in Sicily (Holthuis 1993). In 1831, Millet after studying material from the rivers of the Maine and Loire area (France) thought he found a different species which he described and named *Hippolyte desmarestii*. Joly (1843) stated that Millet erroneously placed the new species in the genus *Hippolyte* Leach, 1814 and transferred it to the genus *Caridina* H. Milne Edwards, 1837. A few years later, de Brito Capello (1867) described a new genus and a species named *Atyaephyra rosiana* from material collected from the surroundings of Coimbra (Portugal) most probably from the River Mondego that crosses the city or from one of its tributaries. Ortman (1890) assigned the species *Caridina desmarestii* to a new genus named *Hemicaridina*. However some years later, he realized that the species *Atyaephyra rosiana* and *Hemicaridina desmarestii* were actually the same and thus proposed a new name combination of this species and established *Atyaephyra desmarestii*.

In the beginning of the 20th century, Bouvier (1913) described two varieties of *Atyaephyra desmarestii*: (a) a westernvariety named *Atyaephyra desmarestii var. occidentalis* Bouvier, 1913, distributed in North Africa up to Tunisia, and the entire area of Southern Europe, up to and including Macedonia; (b) an eastern one, *Atyaephyra desmarestii var. orientalis* Bouvier, 1913, found in Syria. Fifty years later, these two forms were elevated to subspecies level by Holthuis (1961) and since *Atyaephyra desmarestii var. occidentalis* contained the name-bearing type of the species it was re-named to *Atyaephyra desmarestii desmarestii*. A third subspecies, *Atyaephyra desmarestii stankoi*, was described by Karaman (1972) from Doirani Lake which is situated at the borders between Greece and Former Yugoslav Republic of Macedonia (F.Y.R.O.M.). Finally, Al-Adhub (1987) described *Atyaephyra desmarestii mesopotamica* from Shatt Al-Arab River and Hammar Lake (Iraq) thus increasing the number of subspecies to four.

Subsequent studies (Gorgin 1996, Anastasiadou et al. 2004) questioned the validity of these four subspecies based on the observed overlapping in the key characters used to separate them. However, Anastasiadou et al. (2004) stated that given the wide distribution of this species and the degree of isolation of its populations it is likely that a detailed examination of other morphological features could reveal real differences among the various populations of this species.

Recently, Anastasiadou et al. (2006) re-described *Atyaephyra desmarestii* Millet, 1831 after studying specimens from Garrone River (France) and 2 years later they (Anastasiadou et al. 2008) re-validated and re-described *Atyaephyra rosiana* de Brito Cappelo, 1867 based on specimens from São Barnabé River (Odelouca River, Algarve, Portugal).

After examining two mitochondrial genes (COI, 16S) from specimens collected mainly from the western Mediterranean area, Garcia Muñoz et al. (2009) proposed the existence of two species: *Atyaephyra desmarestii*, distributed in West Europe and North Africa and *Atyaephyra stankoi* Karaman, 1972 distributed in Greek freshwaters which was elevated from the subspecies to the species level. Furthermore, the authors argued about the existence of a third genetically distinguished group, *Atyaephyra mesopotamica* Al-Adhub, 1987 (or *Atyaephyra orientalis* Bouvier, 1913), without confirming its status as a distinct species. In addition, they synonomised *Atyaephyra rosiana*, as described by Anastasiadou et al. (2008), with *Atyaephyra desmarestii*. The species *Atyaephyra stankoi* was characterized as cryptic since previous studies failed to detect any distinguishing morphological characters (Anastasiadou et al. 2004) that would enable its discrimination from the *Atyaephyra desmarestii* complex (Garcia Muñoz et al. 2009).

A comprehensive revision of synonyms of the *Atyaephyra*, at species level, has been provided by De Grave and Fransen(2011) while a list of synonyms at genus level is given by Holthuis (1993).

This eventful taxonomic history, and the high intra- and inter-specific morphological variability observed among the *Atyaephyra* taxa make the recognition of discrete species intricate. Also, the wide distribution of the genus and the apparent isolation between populations may support the existence of new non-described species. Therefore the lack of any study including material covering all the known distribution of the genus provoked the present current multidisciplinary study.

In an attempt to recognize and delimit species within *Atyaephyra*, samples covering the known distribution of the genus were analysed, using morphological and molecular methods to evaluate the consensus of groupings as inferred by both datasets. In the last decade molecular data have been widely used in conjunction with decapod morphology, and have been instrumental in discriminating cryptic or sibling species (e.g. Macpherson and Machordom 2005, Jesse et al. 2010, 2011).

This study specifically aims to: (a) test the status of the species already recognized based on morphological and molecular data; (b) describe new species based on morphological and molecular data; (c) provide knowledge on the current geographic distribution of the *Atyaephyra* species; (d) describe the phylogenetic relationships of new and previously described species based on COI gene.

## Material and methods

### Abbreviations used

MMNH: Macedonian Museum of Natural History, Skopje, F.Y.R.O.M.; ZMAUTH: Zoological Museum of the Department of Biology, Aristotle University of Thessaloniki, Greece; MNHN: Muséum National d’Histoire Naturelle, Paris, France; NHM: Natural History Museum, London, England; NMW: Naturhistorisches Museum Wien, Austria; OUMNH: Oxford University Museum of Natural History, England; SMF: Senckenberg Research Institute and Natural History Museum, Frankfurt, Germany and NHMC: Natural History Museum of Crete, Greece; CL: carapace length (measured from the posterior margin of the orbit to the posterior margin of the carapace); stn: station; ovig: ovigerous.

### Morphological analyses

**Figure 1.**
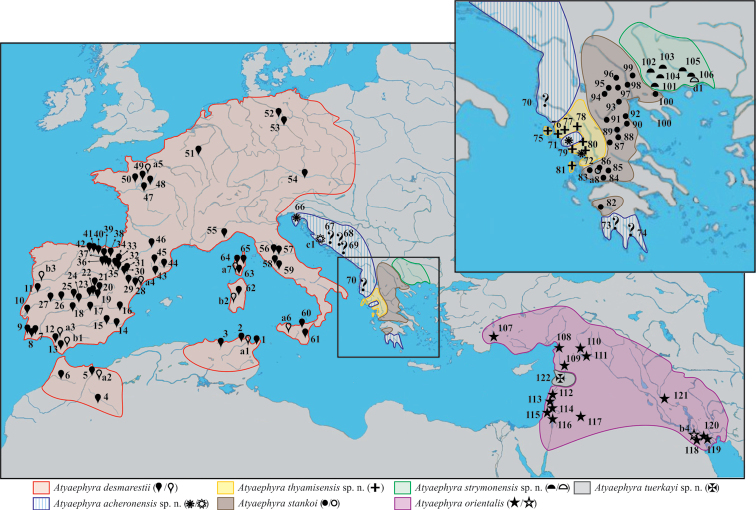
Map showing the sampling localities of *Atyaephyra* and the geographic distribution of the genus in Europe, Middle East and North Africa. Numbers 1–122, next to a solid symbol, indicate the different rivers, lakes or barrages from where samples were collected. Letters a–d, next to an open symbol, represent localities reported in the published sources of sequences. The symbols correspond to different *Atyaephyra* species. Question marks indicate station’s unsure placement inside *Atyaephyra acheronensis* (the clarification of their position will have to await the sequencing) while the general distribution of *Atyaephyra acheronensis* shown is only speculation.

Specimens were collected with a hand dredge over the period 2000–2012 from numerous river catchments in Greece, while additional material from the rest of the Mediterranean region was either offered or loaned by researchers and Museum collections. Samples were loaned or offered from the following museums: NHM, NMW, MNHN, MMNH, ZMAUTH, OUMNH and SMF. In total 1,082 adult individuals (*Atyaephyra acheronensis* sp. n.: 4, *Atyaephyra desmarestii*: 431, *Atyaephyra thyamisensis* sp. n.: 194, *Atyaephyra orientalis*: 111, *Atyaephyra stankoi*: 106, *Atyaephyra strymonensis* sp. n.: 92, *Atyaephyra tuerkayi* sp. n.: 2; furthermore 112 and 30 additional individuals were examined pending their assignment to *Atyaephyra acheronensis* and *Atyaephyra tuerkayi* respectively) were examined from 122 different stations (49 river basins, 20 countries) spanning throughout the known distribution of the genus *Atyaephyra* from Middle East to North Africa and Europe (Fig. 1). Part of this examined material has been included in the studies of Kinzelbach and Koster (1987) and Anastasiadou et al. (2004, 2006, 2008). A total of 135 morphological characters including 68 somatometric distances were analysed (see Appendix: Table 1). Morphometric measurements were taken using a Carl Zeiss standard trinocular microscope or an Olympus VM stereoscope both with ocular micrometer. Only adult individuals were taken into account in order to exclude deviations in the features which appear in the juvenile individuals. A threshold of CL ≥ 5 mm was set for all the specimens examined except for those belonging to *Atyaephyra orientalis* for which the threshold was set to CL ≥ 3.8 mm. The threshold corresponds to the smaller ovigerous individual found. *Atyaephyra orientalis* is of smaller size and thus the threshold must be lower than in the other species. Drawings were made based on photos taken which were subsequently digitized and processed with CorelDRAW® Graphics Suite X5.

### Electronic publication

All data (e.g. taxon descriptions, figures, characters measured) underlying this publication can also be accessed on *Atyaephyra* Scratchpad (http://atyaephyra.myspecies.info/ ). Scratchpads (http://scratchpads.eu ) is a Virtual Research Environment, that enable taxonomists to collaborate in the production of websites documenting the diversity of life (Blagoderov et al. 2010).

### Molecular analyses. DNA extraction, amplification and sequencing

Genomic DNA was extracted exclusively from abdominal tissue using ammonium acetate protocol (provided by Poulakakis N, NHMC, University of Crete, Greece). Abdominal tissue was dissolved in 600μl extraction buffer (0.05M Tris-HCl pH 7.5, 1mM EDTA pH 8.0, 0.15M NaCl, 0.3% sodium dodecyl sulfate, and 0.6μg/μl proteinase K) and incubated in a shaking waterbath at 56°C overnight. Following the incubation, 340μl of 4M ammonium acetate were added to each sample and incubated at room temperature for 60 min. Samples were mixed several times during this period by inversion. The solution was centrifuged at 18,000g for 20 min and supernatant was transferred to 2.0ml centrifuge tubes and 1ml of absolute ethanol was added to each sample. The tubes were inverted several times and centrifuged at 18,000g for 30 min. Following the removal of ethanol samples were dried overnight. DNA pellet was diluted by adding 50μl ddH_2_O and incubated at 4°C overnight. A fragment of the 5' region of mitochondrial (mtDNA) cytochrome c oxidase subunit I (COI) gene was amplified using the polymerase chain reaction (PCR). Two pairs of primers were used for each DNA extract, following the technique of nested PCR. Different combinations of primers were used as first pair: (a) LCO-1490 (5'-GGTCAACAAATCATAAAGATATTGG-3'; Folmer et al. 1994) and HCO-2198 (5'-TAAACTTCAGGGTGACCAAAAAATCA-3'; Folmer et al. 1994); (b) LCO-1490 and C1-N-2191 (5'-CCCGGTAAAATTAAAATATAAACTTC-3'; Simon et al. 1994); (c) Pals-COI-F1 (5'-GAGCTGAACTAGGTCAACC-3', designed on Palaemoninae sequences) and HCO-2198 specifying a ~700 bp to ~600 bp fragment of the COI gene. Thermocycling was performed with an initial denaturation step of two min at 94°C; followed by 35 cycles of one min at 94°C, one min at 42–52°C (depending on the primer pair used), and one min at 72°C, with a final extension of 72°C for 10 min. Then, the primary PCR product was directly used for another amplification reaction, without further purification, using two different combinations of primers as second pair: (a) the newly designed Pals-COI-F1 and Pals-COI-R1 (5'-AGTATAGTAATAGCTCCAGC-3', designed on Palaemoninae sequences) and (b) C1-J-1718, (5'-GGAGGATTTGGAAATTGATTAGTTCC-3'; Simon et al. 1994) and Pals-COI-R1 which amplified a ~450 bp and ~330 bp fragment respectively. The thermal profile for the secondary amplification reaction was the same as that of the primary amplification reaction. All amplification reactions were carried out in a final volume of 20μl. Each reaction contained 1.0μl template DNA, 0.15μM of each primer, 0.15mM dNTPs, 1.5mM or 3mM MgCl2 (depending on the primer pair used), 1X PCR reaction buffer, and 0.5U Taq (Gennaxon).

In some cases after the nested PCR a re-amplification was made using a modified Band-stab PCR protocol (Bjourson and Cooper 1992). The re-amplification reaction was carried out in a final volume of 50μl containing: 0.1μM of each primer, 0.08mM dNTPs, 1mM MgCl2, 1X PCR reaction buffer, and 1.25U Taq (Gennaxon). After an initial denaturation step of two min at 94°C, 25 cycles of one min at 94°C, one min at 45°C, and one min at 72°C were performed, followed by a final extension of five min at 72°C. The amplified fragments were then purified using ethanol and sodium acetate precipitation method and sequenced using Big Dye Terminator Cycle Sequencing 3.1 (Applied Biosystems) standard protocol on an ABI 3730 Genetic Analyzer (Applied Biosystems). All individuals were sequenced either with the forward or the reverse COI primer or with both (Pals-COI-F1, Pals-COI-R1).

### Alignment and genetic divergence

Thirty-seven new COI sequences were generated (GenBank accession numbers JX289898–JX289919, JX289921–JX289933, JX289935–JX289936; Table 1). Our dataset was supplemented with eight COI sequences of *Atyaephyra* from the study of Garcia Muñoz et al. (2009), one from Franjević et al. (2010), one from Zakšek et al. (2007) and four from Page et al. (unpublished data). Furthermore, three COI sequences (Page et al. 2005a, Zakšek et al. 2007, Garcia Muñoz et al. 2009) from another two atyid genera, were included as outgroups (i.e. *Dugastella valentina* (Ferrer Galdiano 1924) from Spain, *Dugastella marocana* Bouvier, 1912 from Morocco, and *Paratya curvirostris* (Heller, 1862) from New Zealand,accession numbers provided in Table 1). The choice of the taxa used as outgroup was based on their close relationship with the genus under study since they all belong to the same atyid group (*Paratya* group) (Von Rintelen et al. 2012).

**Table 1.** *Atyaephyra* specimens and COI sequences accession numbers listed by area and species. The sex and the CL are given for each specimen sequenced in parenthesis (first column). Museum accession numbers are given in parentheses (second column). GenBank accession numbers of published sequences, used in this study, are provided with their corresponding studies indicated by the letters a–e [a: Garcia Muñoz et al. 2009, b: Page et al. (unpub sequences), c: Franjević et al. 2010, d: Zakšek et al. 2007, e: Page et al. 2005a].

Specimen	Sampling site	Station number in Fig. 1	GenBank accession no. COI
*Atyaephyra desmarestii*			
Leb1 (♀, CL: 6.6 mm)	Tunisia, Lebna Barrage, 21.3.2010, coll. S. Dhaouadi-Hassen	1	JX289898
Met1 (♀, CL: 6.8 mm)	Tunisia, Ben Metir Barrage, 22.2.1974 (NHM 1515–1540.22.2.74)	2	JX289899
Moul1 (♀, CL: 6.1 mm)	Morocco, Moulouya River, 11.4.2011, coll. M. Melhaoui	5	JX289900
Krum2 (♀, CL: 6.9 mm)	Morocco, Krumane River, 22.7.1952, coll. J. Phillipson (NHM 1953.12.2.12–15)	6	JX289901
Bord2 (♀, CL: 5.7 mm)	Portugal, Bordeira River, 5.3.1985, coll. J. Paula (NHM 1986.261)	9	JX289902
Sint1 (♀, CL: 7.0 mm)	Portugal, Tagus Basin, Colares River, 1880 (NHM 1880.36)	10	JX289903
Mon1 (♀, CL: 7.2 mm)<br/> Mon2 (♀, CL: 6.8 mm)	Portugal, Mondego Basin, Ceira River, 24.5.2010, coll. V. Ferreira	11	JX289904<br/> JX289905
Vet1 (♀, CL: 8.0 mm)	Spain, Guadalquivir Basin, Guadiamar River, 8.5.2006, coll. C. Lejeusne	12	JX289906
Mu1 (♀, CL: 6.9 mm)	Spain, Segura Basin, Mundo River, 27.9.2001, coll. J.L. Moreno Alcaraz	15	JX289907
Vb1 (♀, CL: 6.1 mm)	Spain, Guadiana Basin, Vado Blanco River, 3.10.2001, coll. J.L. Moreno Alcaraz	17	JX289908
Ta1 (♀, CL: 7.8 mm)	Spain, Tagus Basin, Tajuna River, 7.8.2001, coll. J.L. Moreno Alcaraz	20	JX289909
Er1 (♀, CL: 8.2 mm)	Spain, Ebro Basin, Erro River, 25.5.2007, coll. J. Oscoz	38	JX289910
Fl1 (♂, CL: 5.3 mm)	Spain, Catalan Basin, Fluvia River, 4.2.2005, coll. M.L. Zettler	44	JX289911
Gar2 (♀, CL: 6.0 mm)	France, Garrone River, 25.8.2004, coll. R. Liasko and S. Combes	46	JX289912
Sart1 (♀, CL: 7.0 mm)	France, Loire Basin, Sarthe River, 20.9.2000, coll. P. Noél	48	JX289913
May2 (♀, CL: 5.6 mm)	France, Loire Basin, Mayenne River, 20.9.2000, coll. P. Noél	49	JX289914
Hav1 (♀, CL: 6.3 mm)	Germany, Elbe Basin, Havel River, 26.8.2005, coll. M.L. Zettler	53	JX289915
Dan1 (♀, CL: 7.4 mm)	Austria, Danube River, 8.10.1998, coll. Zipek and Melcher (NMW 18315)	54	JX289916
Sim3 (♀, CL: 6.5 mm)	Sicily, Simeto River, 1.9.1978, coll. C. Froglia	61	JX289917
Riz1 (♂, CL: 5.8 mm)	Corsica, Rizzanese River, 13.8.2003, coll. M.L. Zettler	64	JX289918
Br1 (♀, CL: 7.9 mm)	Corsica, Bravone River, 16.8.2003, coll. M.L. Zettler	65	JX289919
	Tunisia, Medjerda River	a1	FJ594343
	Morocco, Zegzel River	a2	FJ594340
	Spain, Guadalquivir River	a3	FJ594339
	Spain, Ebro River	a4	FJ594342
	France, Loire Basin, Mayenne River	a5	FJ594341
	Sicily, Frattina River	a6	FJ594344
	Corsica, Liamone River	a7	FJ594345
Guad1	Spain, Guadalhorce River, coll. C.N. Sánchez	b1	JX853921
Cog1	Sardinia, Coghinas River, coll. M. Jowers	b2	JX853920
Dour1	Portugal, Douro River, coll. M. Fidalgo	b3	JX289920
*Atyaephyra acheronensis* sp. n.			
Drag1 (♂, CL: 5.1 mm)	Slovenia, Dragonja River, Aug.1971	66	JX289921
Ach1 (♀ ovig., CL: 5.9 mm)	Greece, Acherontas River, 15.4.2012, coll. Ch. Anastasiadou (NHM 2012.1493)	71	JX289922
Lour1 (♀, CL: 7.6 mm)<br/> Lour2 (♀ ovig., CL: 7.0 mm)	Greece, Louros River, 15.4.2012, coll. Ch. Anastasiadou	72	JX289923<br/> JX289924
	Croatia, Krka River	c1	DQ320047
*Atyaephyra thyamisensis* sp. n.			
Lour3 (♀, CL: 7.4 mm)	Greece, Louros River, 15.4.2012, coll. Ch. Anastasiadou	72	JX289925
Lef2 (♂, CL: 5.7 mm)	Greece, Lefkada Island, Vardas River, 2.10.1932, coll. Beier (NHMW 466)	81	JX289926
*Atyaephyra stankoi*			
Doir2 (♀, CL: 5.0 mm)	Greece–F.Y.R.O.M., Doirani Lake, 26.10.1994, coll. S. Jovanovich	99	JX289927
	Greece, Lisimakhia River	a8	FJ594346
*Atyaephyra strymonensis* sp. n.			
Myl1(♀, CL: 5.2 mm)<br/> Myl2 (♀, CL: 5.3 mm)	Greece, Strymonas Basin, Mylopotamos Springs, 23.5.2011, coll. M. Christodoulou and M.S. Kitsos	102	JX289928<br/> JX289929
	Greece, Nestos River	d1	DQ641570
*Atyaephyra orientalis*			
Kar2 (♀, CL: 4.5 mm)	Turkey, Orontes Basin, Karasu River, 22.9.1982, coll. R.K. Kinzelbach (SMF 12174)	108	JX289930
Or2 (♀, CL: 5.0 mm)	Syria, Orontes River, 30/31.3.1979, coll. R.K. Kinzelbach (SMF 12050)	109	JX289931
Euph2 (♀, CL: 4.7 mm)	Syria, Euphrates River, 17.8.1978, coll. R.K. Kinzelbach (SMF 12188)	110	JX289932
Shat2 (♀, CL: 5.3 mm)	Iraq, Euphrates–Tigris Basin, Shatt Al-Arab River, 2011, coll. M.D. Naser	120	JX289933
AlH1	Iraq, Euphrates–Tigris Basin, Al-Huaizah Marshes, coll. M.D. Naser	b4	JX289934
*Atyaephyra tuerkayi* sp. n.			
Nah1 (♀, CL: 6.2 mm)<br/> Nah2(♀, CL: 7.1 mm)	Syria: Nahr Al-Kabir River, 5.3.1979, coll. R.K. Kinzelbach (SMF 43020-1)	122	JX289935<br/> JX289936
Outgroups			
*Dugastella valentina*	Spain	d2	DQ641569
*Dugastella marocana*	Morocco	a9	FJ594347
*Paratya curvirostris*	New Zealand (North Island), Marawara Stream	e1	AY661487

COI sequences were aligned using FSA (Fast Statistical Alignment) (Bradley et al. 2009) and translated into amino acids prior to analysis, to ensure that no gaps or stop codons were present in the alignment. The number of distinct haplotypes was estimated with the software Arlequin version 3.5.1.3 (Excoffier and Lischer 2010). jModelTest (Posada 2008) was used to determine the model of DNA sequence evolution that best fit the data using AIC and BIC criteria. Sequence divergences were estimated with the software MEGA version 5.1 (Tamura et al. 2011).

### Phylogenetic analyses

Phylogenetic inference analyses were conducted using Neighbor Joining (NJ), Maximum Likelihood (ML), and Bayesian Inference (BI) methods. The nucleotide substitution model selected by jModeltest [Tamura-Nei, 1993 (TrN) + gamma (G)] was applied to the data matrix in all analyses. A NJ tree was produced with the software MEGA where branch support was assessed with 1,000 bootstrap replicates. ML estimates were made using PhyML online web server (Guindon et al. 2010; http://www.atgc-montpellier.fr/phyml/ ). Nearest neighbor interchanges (NNIs) and subtree pruning and regrafting (SPR) topological moves were used to explore the space of tree topologies. Approximate likelihood-ratio test (aLRT) based on a non-parametric Shimodaira-Hasegawa-like (SH-like) procedure was employed to estimate branch support (Guindon et al. 2010). BI analysis was performed in BEAST version 1.7.2. (Drummond et al. 2012) assuming an uncorrelated lognormal relaxed-clock model, setting the tree prior to Yule process, run for 100,000,000 generations (10% was discarded as burn-in period). Finally, TreeAnnotator was used to find the Maximum Clade Credibility tree. In order to show the geographic distribution of the distinct haplotypes, in all the analyses, not only the unique haplotypes were used, but all the sequences acquired.

## Results

### Phylogenetic analyses

Out of the 51 *Atyaephyra* COI sequences 35 distinct haplotypes were distinguished. Shared haplotypes were observed among individuals in close geographical proximity. Of the 600 nucleotide sites examined, 237 were variable of which 197 were parsimony informative (14% in the first, 2% in second, and 84% in third codon position). The nucleotide substitution model that best fits our data according to both AIC and BIC criteria is Tamura and Nei (1993) + gamma (G) based on which *Atyaephyra* sequence divergence ranged from 0% to 25.7%.

**Figure 2.**
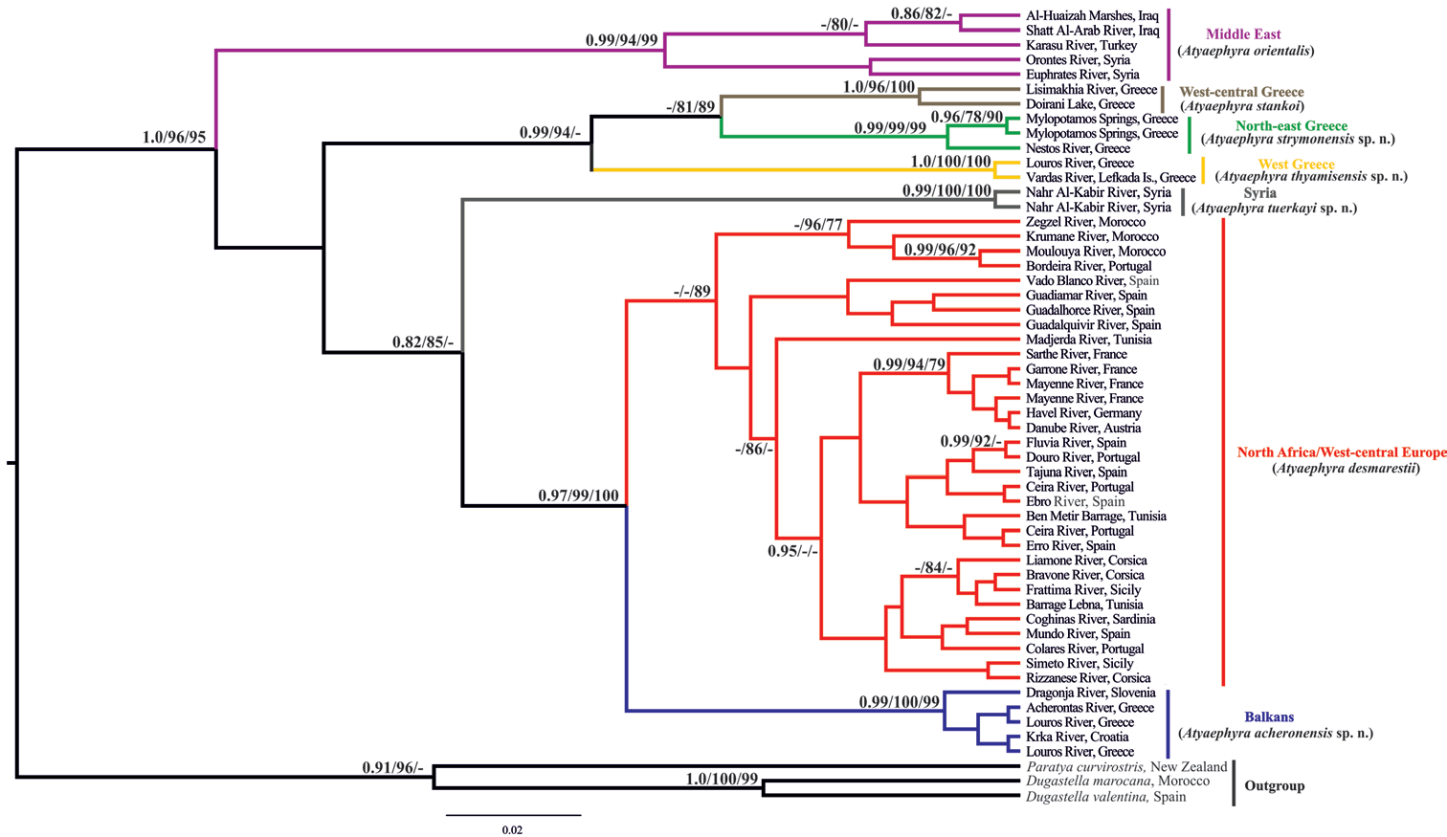
Bayesian inference phylogenetic tree of *Atyaephyra* based on COI dataset. Numbers on nodes indicate Bayesian Inference posterior probabilities, Maximum Likelihood SH-like branch support and Neighbor Joining bootstrap respectively. Only values above 0.75 and 75% are shown. Colours correspond to those used in Figure 1.

All employed methods yielded consistent tree topologies (Fig. 2). The monophyly of the genus is highly supported in all methodologies (BI posterior probability: 1.0, ML SH-like value: 96, NJ bootstrap value: 95).

In all phylogenetic analyses four main and well-supported phylogroups were identified, corresponding to different groups of species designated by morphology (presented in the next section) and/or well defined geographic regions throughout the Mediterranean region (Fig. 2). The first phylogroup comprises specimens from the Middle East which were classified to the nominal species, *Atyaephyra orientalis* by morphology. Specimens from the topotypical populations of the subspecies *Atyaephyra desmarestii orientalis* (Orontes River, Syria) and *Atyaephyra desmarestii mesopotamica* (Shatt Al-Arab River, Iraq) were also included. However, present data do not allow for within clade fine scale resolution. The mean genetic distances between the Middle East phylogroup (*Atyaephyra orientalis*) and the other groups/subgroups were very high ranging from 18.7% to 24.5% while the average intraspecific distance was 5.8% (Table 2).

**Table 2.** Nucleotide mean distances (% Tamura-Nei 1993 + G model) of cytochrome c oxidase I (COI) within (first column) and among the *Atyaephyra* species. The range of pairwise distances is given in parenthesis.

	Within species	*Atyaephyra desmarestii*	*Atyaephyra acheronensis* sp. n.	*Atyaephyra thyamisensis* sp. n.	*Atyaephyra stankoi*	*Atyaephyra strymonensis* sp. n.	*Atyaephyra orientalis*
*Atyaephyra desmarestii*	0.016<br/> (0.000–0.048)						
*Atyaephyra acheronensis* sp. n.	0.001<br/> (0.000–0.003)	0.083<br/> (0.059–0.116)					
*Atyaephyra thyamisensis* sp. n.	0.000<br/> (0.000)	0.239<br/> (0.206–0.271)	0.238<br/> (0.233–0.251)				
*Atyaephyra stankoi*	0.024<br/> (0.024)	0.236<br/> (0.204–0.261)	0.232<br/> (0.215–0.241)	0.167<br/> (0.163–0.176)			
*Atyaephyra strymonensis* sp. n.	0.003<br/> (0.000–0.005)	0.233<br/> (0.201–0.273)	0.219<br/> (0.205–0.234)	0.182<br/> (0.166–0.194)	0.119<br/> (0.117–0.119)		
*Atyaephyra orientalis*	0.058<br/> (0.009–0.102)	0.222<br/> (0.192–0.287)	0.238<br/> (0.216–0.256)	0.187<br/> (0.169–0.200)	0.226<br/> (0.190–0.244)	0.245<br/> (0.219–0.270)	
*Atyaephyra tuerkayi* sp. n.	0.000<br/> (0.000)	0.230<br/> (0.208–0.260)	0.222<br/> (0.215–0.232)	0.257<br/> (0.237–0.278)	0.232<br/> (0.215–0.242)	0.254<br/> (0.243–0.267)	0.197<br/> (0.172–0.221)

The second phylogroup which is strongly supported by both BI and ML methodologies while in NJ yielded lower bootstrap values (BI posterior probability: 0.99, ML SH-like value: 94, NJ bootstrap value: 65) includes sequences exclusively from Greek populations. The Greek phylogroup is further subdivided into three well supported groups. The first subgroup corresponds to the nominal species, *Atyaephyra stankoi*, found in West-central Greece. It is worth noticing that specimens from the type locality (Doirani Lake) of *Atyaephyra desmarestii stankoi* are also included. The remaining Greek specimens are grouped in two well defined subgroups, one distributed in North-east Greece while the other is located in West Greece (Fig. 1). The mean genetic divergence among the three subgroups ranges from 11.9% to 18.2%, while the mean genetic distances within subgroups varied from 0% to 2.4% (Table 2).

The third phylogroup contains specimens from the Syrian River Nahr Al-Kabir and it is strongly supported in all methodologies (BI posterior probability: 0.99, ML SH-like value: 100, NJ bootstrap value: 100). The mean genetic distances between the Syrian subgroup and the other groups/subgroups were very high ranging from 19.7% to 25.7% (Table 2).

The fourth phylogroup which is well supported by BI, ML and NJ (BI posterior probability: 0.97, ML SH-like value: 99, NJ bootstrap value: 100) includes specimens originating from West-central Europe, North Africa and the Balkans. Within this phylogroup, specimens from Croatia, Slovenia and Greece form a distinct highly supported subgroup (BI posterior probability: 0.99, ML SH-like value: 100, NJ bootstrap value: 99). The remaining specimens within the phylogroup i.e. specimens from West-central Europe and North Africa, although classified as *Atyaephyra desmarestii* (nominal species) by morphology (discussed in the next section) do not constitute a well supported subgroup except in NJ analysis where it is relatively well supported (NJ bootstrap value: 89). Sequences from the topotypical populations of the *Atyaephyra desmarestii* (Mayenne and Sarthe River), and *Atyaephyra rosiana* described by de Brito Capello (Ceira River, tributary of Mondego River) were included in this subgroup as well as a sequence acquired from river Bordeira (Portugal) which is near to São Barnabé River from where *Atyaephyra rosiana* was re-described by Anastasiadou et al. (2008). The genetic distances between these two subgroups are quite large, ranging from 5.9% to 11.6% (Table 2). The lowest values (5.9–6.8%) were observed between the specimens of the Balkan subgroup and those of South Iberian Peninsula and North Africa (Morocco), located in the distant end of *Atyaephyra desmarestii* distribution. On the contrary higher values (7.5–10.2%) were observed between the nearest to the Balkan subgroup populations (e.g. Danube River) as well as between the topotypical populations of *Atyaephyra desmarestii* (Mayenne and Sarthe River) and the Balkan populations. Furthermore, no haplotypes were shared between these two subgroupings.

### Morphological analysis. Account of *Atyaephyra* species

The present study recognises five well defined by morphology species of *Atyaephyra*: *Atyaephyra desmarestii* (Millet, 1831), *Atyaephyra stankoi* Karaman, 1972, *Atyaephyra orientalis* Bouvier, 1913 and two new species, *Atyaephyra thyamisensis* sp. n. and *Atyaephyra strymonensis* sp. n. Neotypes are designated for *Atyaephyra desmarestii* and *Atyaephyra stankoi* in an attempt to stabilize their taxonomy. In addition, two cryptic species are defined by the molecular analysis. Descriptions are provided for all these species.

## Taxonomy

### Family Atyidae de Haan, 1849 (in de Haan, 1833–1850)

#### Genus Atyaephyra

de Brito Capello, 1867

http://species-id.net/wiki/Atyaephyra

##### Type species:

*Atyaephyra rosiana* de Brito Capello, 1867: 6–7, Pl. 1, Figs 1A–E [type locality: Coimbra, Portugal]; by monotypy.

##### Diagnosis.

Carapace with supraorbital and antennal tooth. Rostrum long and armed up to the tip. Eyes well developed, pigmented. Exopods present only on the two first pairs of pereiopods, carpus of first and second pair of pereiopods with a distal excavation. Uropod diaeresis with a single spine (rarely two). Appendix masculina of male second pleopod long, sub-cylindrical and armed with numerous spiniform setae. Eggs small to medium, size 0.40–0.75 × 0.25–0.5 mm.

#### Atyaephyra desmarestii

(Millet, 1831)

http://species-id.net/wiki/Atyaephyra_desmarestii

**Figure 3.**
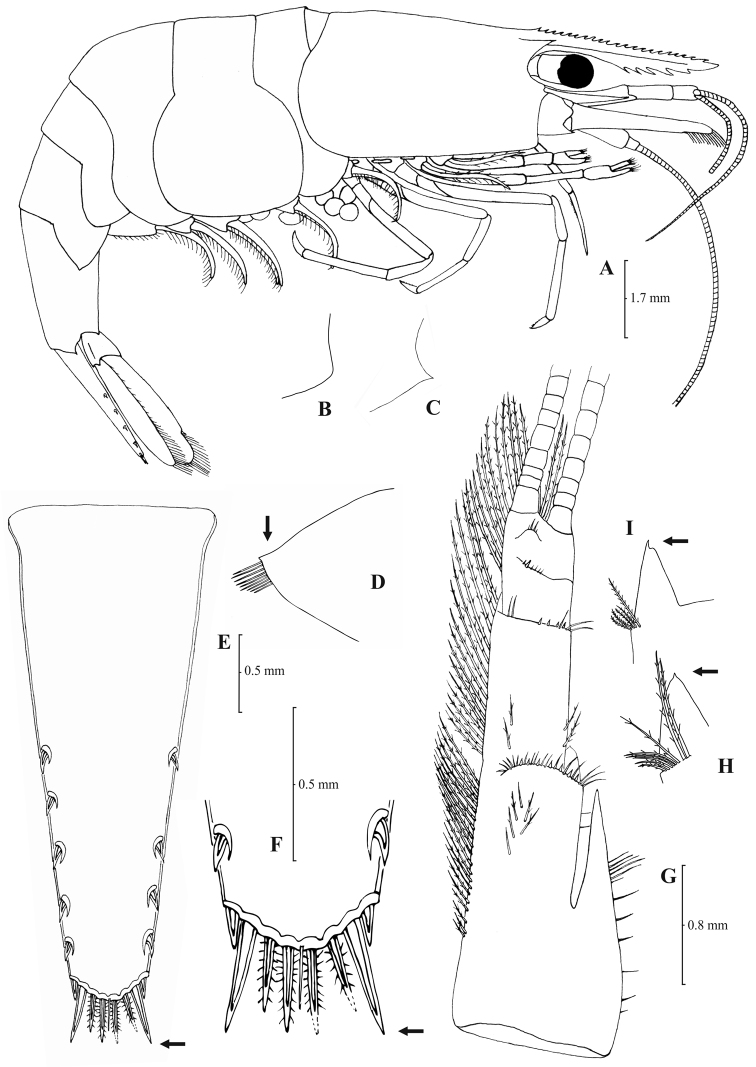
*Atyaephyra orientalis* Bouvier, 1913, adult ovig. ♀ (SMF 12050): **A** entire individual **B** detail ofpterygostomial boarder **C** detail ofpterygostomial boarder (adult ♀, SMF 12050) **D** right pleuron of fifth abdominal segment **E** telson **F** distal margin of telson **G** right antennular peduncle **H** right antennular lobe **I** right antennular lobe (adult ♀, SMF 12050).

Symethus fluviatilis Rafinesque, 1814: 23–24 [suppressed under the plenary powers for the purposes of the Principle of Priority but not for those of the Principle of Homonymy in Opinion 522 in 1958].
Acilius fluviatilis . –Rafinesque 1815: 221.
Hippolyte Desmarestii Millet, 1831: 55–57, Pl. 1, Figs 1A–B [type locality: Mayenne River, Sarthe River, Loir River, Thouet River, Layon River (France)]. –H. Milne Edwards 1837: 376; Taramelli 1864: 363–369.
Caridina Desmarestii . –Joly 1843: 34–86, Figs 1–78; Heller 1863: 238, Pl. 8, Fig. 3; Pelseneer 1886: 211–216; Bolivar 1892: 131.
Atyaephyra Rosiana de Brito Capello, 1867: 6–7, Pl. 1, Figs 1A–E. [type locality: Coimbra, Portugal].
Hemicardina desmarestii . –Ortmann 1890: 464–465.
Atyaëphyra Desmaresti . –Ortmann 1895: 401; Bouvier 1925: 84–89, Figs 164–174, partim.
Atyaëphyra Desmaresti var. *occidentalis* Bouvier, 1913: 65–74, Figs 2E–H, 2J–L, 3E–J, partim.
Atyaephyra desmarestii desmarestii . –Holthuis 1961: 5–10, Figs 2A, 3A, partim.
Atyaephyra desmarestii . –Anastasiadou et al. 2004: 5–13, partim; Anastasiadou et al. 2006: 1195–1207, Figs 1–5; Garcia Muñoz et al. 2009: 32–42; Von Rintelen et al. 2012: 82–96, partim.
Atyaephyra rosiana . –Anastasiadou et al. 2008: 191–205, Figs 1–5.

##### Material examined.

**Type material.** Neotype: 1 ovig. ♀ (CL 7.1 mm), MNHN-IU-2009-2270 (ex MNHN-Na480), Maine-et-Loire, France [here designated].

##### Non-type material.

**Tunisia:** 8 ♀♀ (1 ovig.) (CL 5.4–7.4 mm), Barrage Lebna (Fig. 1, stn 1), 21.3.2010, coll. S. Dhaouadi-Hassen; 2 ♀♀ (CL 6.0–6.8 mm), NHM 1515–1540.22.2.74, Ain Draham, Barrage Ben Metir (Fig. 1, stn 2), 22.2.1974. **Algeria:** 1 ♂ (CL 5.1 mm), NHM 1955.5.3.15–18, Algiers, Seybouse River (Fig. 1, stn 3), 3.5.1955; 11 ♀♀ (6 ovig.) (CL 5.0–8.0 mm) and 1 ♂ (CL 5.2 mm), NHM 1949.5.2.1–12, Beni Abbes, Saoura River (Fig. 1, stn 4), 2.5.1949, coll. H. Munro Fox. **Morocco:** 4 ♀♀ (1 ovig.) (CL 5.5–6.5 mm) and 1 ♂ (CL 5.0 mm), Moulouya River (Fig. 1, stn 5), 11.4.2011, coll. M. Melhaoui; 1 ♀ (CL 6.9 mm) and 4 ♂♂ (CL 5.2–5.6 mm), NHM 1953.12.2.12–15, Krumane River (Fig. 1, stn 6), 22.7.1952, coll. J. Phillipson. **Portugal:** 21 ♀♀ (12 ovig.) (CL 5.8–7.3 mm) and 11 ♂♂ (CL 5.0–5.7 mm), Algarve, São Barnabé River (Odelouca River) (Fig. 1, stn 7), 23.7.1988, coll. C. d' Udekem d' Acoz; 7 ♀♀ (6 ovig.) (CL 6.2–7.7 mm) and 5 ♂♂ (CL 5.0–5.2 mm), NHM 1971.105, Portimao, Odelouca River (Fig. 1, stn 8), 1970; 18 ♀♀ (4 ovig.) (CL 5.5–8.0 mm) and 3 ♂♂ (CL 5.0–5.1 mm), NHM 1986.261, Bordeira River (Fig. 1, stn 9), 5.3.1985, coll. J. Paula; 5 ♀♀ (4 ovig.) (CL 7.0–8.1 mm) NHM 1880.36, Sintra, Colares River (Fig. 1, stn 10), 1880; 15 ♀♀ (3 ovig.) (CL 5.8–7.9 mm) and 5 ♂♂ (CL 5.3–6.1 mm), Coimbra, Ceira River (Fig. 1, stn 11), 24.5.2010, coll. V. Ferreira. **Spain:** 2 ♀ (CL 6.5–8.0 mm), Veta la Arena, Guadiamar River (Fig. 1, stn 12), 8.5.2006, coll. C. Lejeusne; 5 ♀♀ (CL 6.1–6.7 mm) and 17 ♂♂ (CL 5.0–6.5 mm), Cadiz, Guadalete River (Fig. 1, stn 13), 2000, coll. A. Rodriguez; 3 ♀♀ (CL 5.1–6.3 mm), Segura River (Fig. 1, stn 14), 28.9.2001, coll. J.L. Moreno Alcaraz; 10 ♀♀ (1 ovig.) (CL 6.1–7.5 mm) and 1 ♂ (CL 5.5 mm), Mundo River (Fig. 1, stn 15), 18/27.9.2001, coll. J.L. Moreno Alcaraz; 2 ♀♀ (CL 6.6–7.7 mm) and 1 ♂ (CL 5.5 mm), Villalva de la Sierra, Jucar River, 40°07.99'N, 02°08.38'W (Fig. 1, stn 16), 16.8.2001, coll. J.L. Moreno Alcaraz; 7 ♀♀ (CL 5.1–6.4 mm) and 1 ♂ (CL 5.3 mm), Ossa de Montiel, Vado Blanco River, 38°54.60'N, 02°48.03'W (Fig. 1, stn 17), 3.10.2001, coll. J.L. Moreno Alcaraz; 3 ♀♀ (CL 5.7–6.5 mm), El Torno, Bullaque River, 39°14.36'N, 04°15.57'W (Fig. 1, stn 18), 11.10.2001, coll. J.L. Moreno Alcaraz; 2 ♀♀ (CL 7.2–7.7 mm), Canavera, Guadiella River, 40°25.36'N, 02°28.95'W (Fig. 1, stn 19), 14.8.2001, coll. J.L. Moreno Alcaraz; 3 ♀♀ (CL 6.2–8.0 mm), Abanades, Tajuna River (Fig. 1, stn 20), 7.8.2001, coll. J.L. Moreno Alcaraz; 3 ♀♀ (1 ovig.) (CL 6.3–7.2 mm) and 6 ♂♂ (CL 5.5–6.5 mm), Henares River, (Fig. 1, stn 21), 1.8.2001, coll. J.L. Moreno Alcaraz; 1 ovig. ♀ (CL 7.4 mm), Naharros, Canamares River, 41°09.10'N, 02°55.14'W (Fig. 1, stn 22), 30.7.2001, coll. J.L. Moreno Alcaraz; 2 ovig. ♀♀ (CL 7.3–7.8 mm), Puebla de Valles, Jarama River (Fig. 1, stn 23), 31.7.2001, coll. J.L. Moreno Alcaraz; 1 ♀ (CL 5.9 mm) and 1 ♂ (CL 5.1 mm) La Guardia, Cedron River, 39°48.26'N, 03°20.33'W (Fig. 1, stn 24), 6.9.2001, coll. J.L. Moreno Alcaraz; 1 ♀ (CL 5.2 mm), Escalona, Alberche River, 40°09.45'N, 04°25.04'W (Fig. 1, stn 25), 27.8.2001, coll. J.L. Moreno Alcaraz; 1 ♀ (CL 5.1 mm) and 2 ♂♂ (CL 5.3–5.7 mm), Tietar River (Fig. 1, stn 26), 28.8.2001, coll. J.L. Moreno Alcaraz; 9 ♀♀ (1 ovig.) (CL 5.1 mm) and 1 ♂ (CL 5.0 mm), Tagus River (Fig. 1, stn 27), 14.8.2001 and 5.9.2001, coll. J.L. Moreno Alcaraz; 1 ♂ (CL 5.5 mm), Calanda, Guadalope River (Fig. 1, stn 28), 25.5.2004, coll. J. Oscoz; 1 ♀ (CL 7.2 mm) and 1 ♂ (CL 5.1 mm), Escatron, Martin River (Fig. 1, stn 29), 24.5.2001, coll. J. Oscoz; 1 ♀ (CL 5.6 mm) and 3 ♂♂ (CL 5.3–5.6 mm), Murillo de Gallego, Gallego River (Fig. 1, stn 30), 7.8.2007, coll. J. Oscoz; 1 ovig. ♀ (CL 6.5 mm), Gurrea de Gallego, Soton River (Fig. 1, stn 31), 14.6.2006, coll. J. Oscoz; 1 ♂ (CL 6.2 mm), Lumbier, Irati River (Fig. 1, stn 32), 8.7.2005, coll. J. Oscoz; 2 ovig. ♀♀ (CL 6.9–7.5 mm) and 4 ♂♂ (CL 5.2–5.8 mm), Aspurz, Salazar River (Fig. 1, stn 33), 3.7.2007, coll. J. Oscoz; 1 ovig. ♀ (CL 6.5 mm) and 1 ♂ (CL 5.2 mm), Ripodas, Areta River (Fig. 1, stn 34), 3.7.2007, coll. J. Oscoz; 5 ♀♀ (4 ovig.) (CL 5.0–7.5 mm) and 2 ♂♂ (CL 5.6 mm), Castejon, Alfaro, Tudela, Ebro River (Fig. 1, stn 35), 11/12.7.2007, coll. J. Oscoz; 6 ♀♀ (5 ovig.) (CL 7.0–8.6 mm), San Adrian, Ega River (Fig. 1, stn 36), 27.6.2007, coll. J. Oscoz; 1 ovig. ♀ (CL 7.3 mm) and 2 ♂♂ (CL 5.2–5.5 mm), Marcilla, Aragon River (Fig. 1, stn 37), 28.6.2007, coll. J. Oscoz; 2 (1 ovig.) ♀♀ (CL 8.2–8.5 mm) and 2 ♂♂ (CL 5.6–6.5 mm), Urroz, Erro River (Fig. 1, stn 38), 25.5.2007, coll. J. Oscoz; 1 ovig. ♀ (CL 7.5 mm) and 2 ♂♂ (CL 5.8–6.0 mm), Mendigorria, Salado River (Fig. 1, stn 39), 14.6.2007, coll. J. Oscoz; 1 ovig. ♀ (CL 7.6 mm), Puentelarreina, Arga River (Fig. 1, stn 40), 20.6.2007, coll. J. Oscoz; 1 ♀ (CL 7.2 mm), Iraneta, Arakil River (Fig. 1, stn 41), 20.6.2007, coll. J. Oscoz; 1 ♀ (CL 7.4 mm), Palazuelos, Jerea River (Fig. 1, stn 42), 1.6.2004, coll. J. Oscoz; 3 ovig. ♀♀ (CL 7.3–8.0 mm) and 2 ♂♂ (CL 5.3–5.5 mm), NHM 1955.10.5.2–6 and NHM 1957.8.12.69–75, Barcelona, Llobregat River (Fig. 1, stn 43), 5.10.1955 and 12.8.1955; 8 ♂♂ (CL 5.2–6.1 mm), Bascara, Fluvia River (Fig. 1, stn 44), 4.2.2005, coll. M.L. Zettler; 3 ♀♀ (CL 5.6–6.6 mm), NHM 1955.10.5.8–10, Gerona, Lake of Banyoles (Fig. 1, stn 45), 5.10.1955. **France:** 30 ♀♀ (18 ovig.) (CL 5.0–7.0 mm) and 20 ♂♂ (CL 5.0–5.2 mm), Merville, Garrone River (Fig. 1, stn 46), 25.8.2004, coll. R. Liasko and S. Combes; 2 ♀♀ (CL 5.5–6.5 mm), NHM 1955.5.3.11–14, Maine et Loire, Loire River (Fig. 1, stn 47), 3.5.1955; 2 ♀♀ (CL 6.6–7.0 mm), Angers, Sarthe River (Fig. 1, stn 48), 20.9.2000, coll. P. Noél; 2 ♀♀ (CL 5.1–5.6 mm), Mayenne River (Fig. 1, stn 49), 20.9.2000, coll. P. Noél; 3 ♀♀ (CL 6.3–6.5 mm), NMW 467, Rennes, Vilaine River (Fig. 1, stn 50), coll. G. Laponge. **Belgium:** 31 ♀♀ (8 ovig.) (CL 5.2–8.3 mm) and 7 ♂♂ (CL 5.0–6.0 mm), Ombret, Meuse River, (Fig. 1, stn 51), 3.8.1979, coll. C. d' Udekem d' Acoz. **Germany:** 1 ♂ (CL 5.2 mm) Berlin, Tegel Lake, 52°34.98'N, 13°16.44'E (Fig. 1, stn 52), 13.9.1995, coll. K. Rudolph and M.L. Zettler; 4 ♀♀ (CL 5.7–7.0 mm) and 1 ♂ (CL 5.0 mm), Havel River (Fig. 1, stn 53), 52°23.82'N, 12°17.04'E, 26.8.2005 (Saxony–Anhalt) and 52°29.82'N, 12°24.30'E, 27.8.2005 (Brandenburg), coll. M.L. Zettler. **Austria:** 1 ♀ (CL 7.4 mm), NMW 18315, Danube River (Fig. 1, stn 54), 8.10.1998, coll. Zipel and Melcher. **Italy:** 2 ♂♂ (CL 5.0–5.7 mm), Centa River (Fig. 1, stn 55), 28.5.1989, coll. C. Froglia; 4 ♀♀ (CL 5.3–5.8 mm) and 1 ♂ (CL 5.6 mm), Nestore River (Fig. 1, stn 56), 11.11.1974, coll. C. Froglia; 2 ♂♂ (CL 5.2–5.6 mm), Ponte Nuovo, Chiascio River, (Fig. 1, stn 57), 9.9.1975, coll. Cianficoni; 2 ovig. ♀♀ (CL 7.0–7.5 mm) and 1 ♂ (CL 5.2 mm), Nera River (Fig. 1, stn 58), 5.6.1971, coll. Moretti; 5 ♀♀ (CL 6.2–6.8 mm) and 7 ♂♂ (CL 5.0–6.3 mm), Tiber River (Fig. 1, stn 59), 10.10.1975 (Nestore), 14.10.1975 (Orte), 13.11.1975 (Umbertide), coll. Cianficoni. **Sicily:** 1 ovig. ♀ (CL 7.5 mm) and 4 ♂♂ (CL 5.4–5.9 mm), San Bartolomeo, Rosmarino River (Fig. 1, stn 60), 13.5.1986, coll. C. Froglia; 2 ♀♀ (CL 5.8–6.4 mm) and 1 ♂ (CL 5.5 mm), Simeto River (Fig. 1, stn 61), 1.9.1978, coll. C. Froglia. **Sardinia:** 7 ♀♀ (4 ovig.) (CL 5.5–7.2 mm) and 2 ♂ (CL 5.0 mm), unknown locality (Fig. 1, stn 62), 13.9.1977, coll. Cav; 2 ♀ (CL 6.7–7.6 mm) and 1 ♂ (CL 5.6 mm), unknown locality, coll. R.B. Manning. **Corsica**: 3 ♀♀ (1 ovig.) (CL 6.3–6.9 mm) and 1 ♂♂ (CL 5.0 mm), Favello, Taravo River (Fig. 1, stn 63), 10.8.2003, coll. M.L. Zettler; 5 ♂♂ (CL 5.0–5.8 mm), Propriano, Rizzanese River (Fig. 1, stn 64), 13.8.2003, coll. M.L. Zettler; 2 ♀♀ (CL 7.2–7.9 mm) and 4 ♂♂ (CL 5.3–6.0 mm), Bravone, Bravone River, 42°12.36'N, 09°32.10'E (Fig. 1, stn 65), 16.8.2003, coll. M.L. Zettler.

##### Amendments to description.

**Figure 4.**
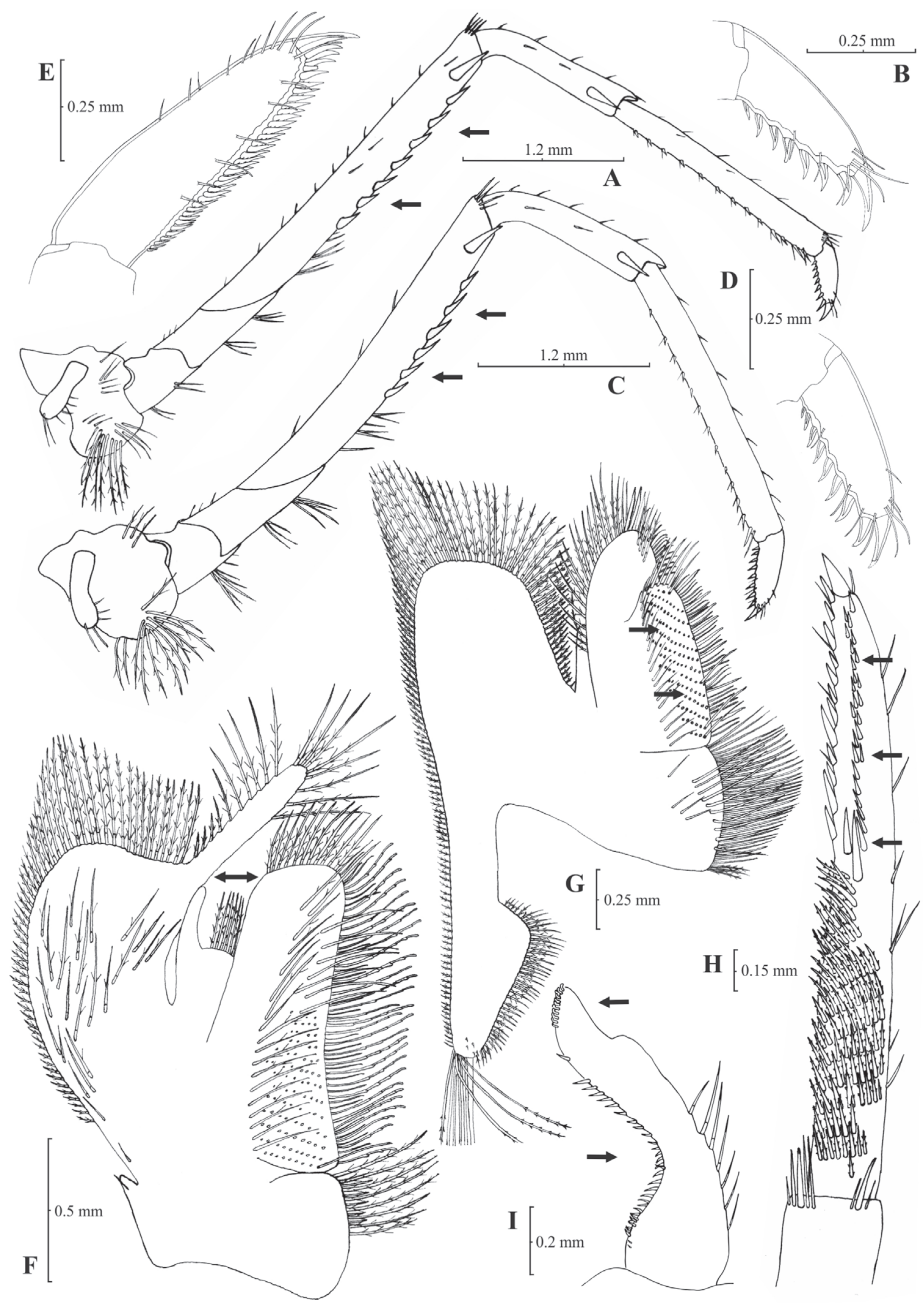
*Atyaephyra orientalis* Bouvier, 1913, adult ovig. ♀ (SMF 12050): **A** right third pereiopod **B** dactylus of third pereiopod **C** right fourth pereiopod **D** dactylus of fourth pereiopod **E** dactylus of right fifth pereiopod **F** right first maxilliped **G** right maxilla **H** rightterminal segment of third maxilliped. Adult ♂ (SMF 12050): **I** right endopod of first male pleopod.

**Figure 5.**
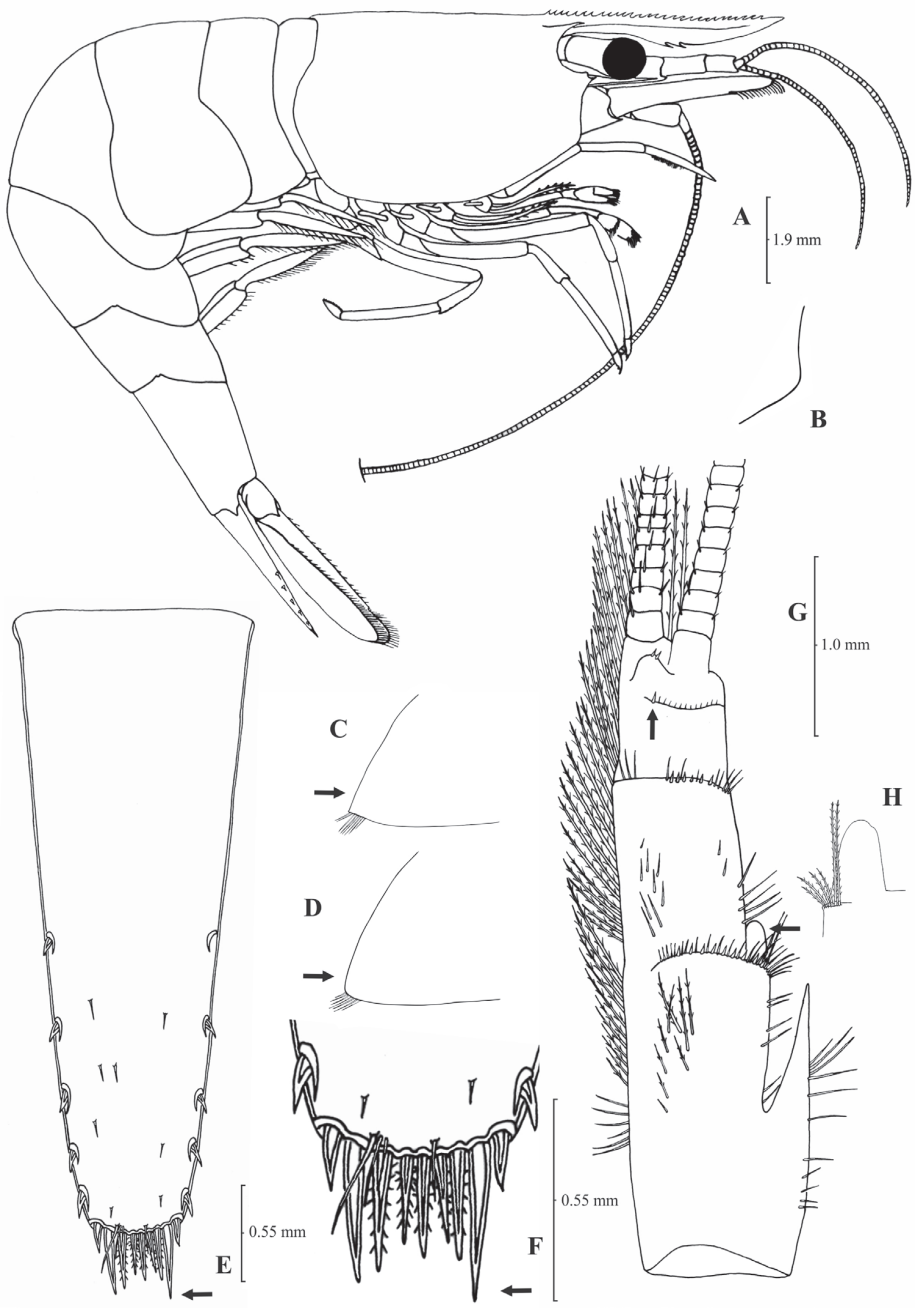
*Atyaephyra stankoi* Karaman, 1972. Neotype, adult ♀ (NHM 2012.1475): **A** entire individual **B** right detail ofpterygostomial boarder **C** right pleuron of fifth abdominal segment **D** right pleuron of fifth abdominal segment (adult ♀) **E** telson **F** distal margin of telson **G** right antennular peduncle **H** right antennular lobe.

Rostrum long, dorsal margin straight or slightly curved in the middle and pointed upwards, 3.79–8.70, mostly (82% of the individuals examined) 4.64–6.50, × as long as high, shorter, equal to, or longer than scaphocerite. From 17 to 36 (21–28 in 86% of the individuals examined) pre orbital teeth on dorsal margin of rostrum arranged to tip. One to five, most frequently (90% of the individuals examined) 2–4, post orbital teeth and 1–13, most often (88% of the individuals examined) 4–9, teeth on ventral margin of rostrum. Carapace smooth with pterygostomial angle not protruding, rounded (Anastasiadou et al. 2006; Fig. 1). Pleuron of fifth abdominal segment pointed with an acute posterior angle. Telsonwith 2–4, most frequently (95% of the individuals examined) 3–4, pairs of dorsal spines arranged in curved fashion. Distal border of telson with 7–15, mostly (89%) 9–13, spines (4–7 pairs) arranged in a fan-like way. Outermost pair of spines shortest, similar to dorsal spines, adjacent pair stronger, terminating before the inner, finely setulose pairs (Anastasiadou et al. 2006; Figs 2A–B). Antennulary stylocerite with its tip failing to reach, reaching or overreaching distal margin of basal peduncle segment. Anterolateral lobe of basal segment short, round or pointed. Distal segment of antennular peduncle with 0–2, predominantly (93%) 1–2, spines (Anastasiadou et al. 2006; Fig. 2D). Basal lower endite of maxilla densely covered with long simple setae arranged in 15–22, mostly (84%) 17–20, oblique parallel rows. Endite of maxilla 1.39–1.88, most often (90%) 1.49–1.71, × as long as basal lower endite (Anastasiadou et al. 2006; Fig. 3C). Basal endite of first maxilliped reaching clearly beyond distal end of exopod (Anastasiadou et al. 2006; Fig. 3D). Distal one-third of terminal segment of third maxilliped bearing 0–8, (1–6 in 91% of the individuals examined), mesial spines and one subdistal lateral spine near the base of larger terminal spine, interpretable as dactylus (Anastasiadou et al. 2006; Fig. 3G). Armature along flexor margin of dactylus of third and fourth pereiopod consisting of 5–10 (6–8 in 95% of the individuals) and 5–10 (6–8 in 94% of the individuals) spines respectively. Merus of third and fourth pereiopod with 1–7 (3–5 in 95% of the individuals) and 2–6 (3–5 in 99% of the individuals) spines respectively (Anastasiadou et al. 2006; Figs 4C–D). Armature along flexor margin of dactylus of fifth pereiopod consisting of 18–43, mostly (87%) 25–35, spines (Anastasiadou et al. 2006; Figs 4E–F). Endopod of first male pleopod expanded proximally and with a distal portion elongated and tapering, often with a small protruding lobe in its outer subdistal part. Endopod with 14–30 (16–25 in 86% of the individuals examined), spines arranged on a slightly curved inner margin and 9–17 (10–15 in 92% of the individuals examined), setae arranged on outer margin (Anastasiadou et al. 2006; Fig. 5C, Anastasiadou et al. 2008; Fig. 5C). 133–848 eggs of 0.4–0.7 × 0.25–0.4 mm size.

##### Size.

*Atyaephyra desmarestii* is a large sized species with maximum carapace length to be 6.8 mm in ♂♂, 8.5 mm in ♀♀ and 8.6 mm in ovig. ♀♀.

##### Molecular characters.

*Atyaephyra desmarestii* can be differentiated from all other species of *Atyaephyra* by molecular characters, as demonstrated by the phylogenetic analysis of mtDNA COI sequences. Furthermore, 22 haplotypes from 30 different localities found in *Atyaephyra desmarestii* were not shared by any other species of the genus. Finally, it differs from all the other species in the following nucleotide positions in the COI gene of *Atyaephyra desmarestii* specimen Dour1 (Genbank accession number JX289920), position 213: cytosine (C), position 234: cytosine (C) and position 444: adenine (A).

##### Distribution.

*Atyaephyra desmarestii* is found in freshwater habitats of North Africa and West-central Europe (see material examined and Fig. 1).

##### Remarks.

*Atyaephyra desmarestii* has been exhaustively described and illustrated by Anastasiadou et al. (2006). Anastasiadou et al. (2008) also re-established and redescribed in detail *Atyaephyra rosiana*, a species currently considered as a synonym of *Atyaephyra desmarestii*. In the present paper the same material used for the redescriptions of *Atyaephyra desmarestii* and of *Atyaephyra rosiana* (Anastasiadou et al. 2006, 2008) was examined. Although Anastasiadou et al. (2006) stated that the “holotype” of *Atyaephyra desmarestii* could not be traced in French institutions, Bouvier (1913) clearly stated that he examined material from “*Maine-et-Loire (H. Milne Edwards, probablement des cotypes de Millet)*”. As Millet and H. Milne Edward were contemporary, and it seemed possible that H. Edwards may have asked for some specimens from the MNHN, this material was recently looked for in the MNHN collection, where the material listed in Bouvier (1913) is indeed still present (registration number Na480). However, there appears to be a discrepancy (and thus possible clarification) on the actual specimen label to this information. The specimen label (see Appendix: Fig. 3) provides the following information: (1) “Maine et Loire”, (2) “*Caridina Desmarestii* Millet”, (3) “A. Milne Edwards det.”, (4) “E.L. Bouvier ver. 1899” and (5) “A. Milne Edwards, 1900”. It is difficult to definitively interpret the label information in view of what Bouvier (1913), a contemporary of A. Milne-Edwards, wrote, as he may have had access to direct, personal information. However, the sample is herein interpreted as having belonged to the A. Milne-Edwards collection, who died in 1900 (1835–1900) and was then accessioned in the museum collection (label item 5), with the material being examined and verified, i.e “*ver*.” in 1899, by Bouvier (label item 4), but that the material originally was identified by A. Milne Edwards (label item 3), and that the material may not have been seen by H. Milne Edwards (although it may have passed from father to son without being recorded as such on the museum labels). It seems, therefore, impossible to certify that these are indeed syntypic specimens of *Hippolyte Desmarestii* Millet, 1831, as indicated by Bouvier (1913). However, in deference to Bouvier’s potential knowledge on the matter and in line with Recommendation 75A (ICZN, 1999), a neotype for *Atyaephyra desmarestii* is herein selected from this lot, the largest ovigerous female. The designation of a neotype is deemed justified under Art. 75 (ICZN, 1999), as (1) the taxon is involved in a complex nomenclatorial problem which cannot be solved without fixing the identity of the oldest name; (2) the taxon is differentiated from the other taxa in this complex by having 0–8 mesial spines on terminal segment of third maxilliped, the basal endite of first maxilliped clearly reaching beyond distal end of exopod, having 1–5 post orbital rostral teeth, having a not protruding, rounded pterygostomial angle and by the slightly curved endopod of first male pleopod with its distal part elongated and tapering; (3) the selected specimen is the largest (of only two) ovigerous females in lot MNHN-Na480; (4) the reasons the name-bearing types are considered lost (or the contrary cannot be conclusively proven) are given above (see also Anastasiadou et al. 2006); (5) the neotype is from the general locality (Maine et Loire) of the type locality of *Atyaephyra desmarestii* from which no other species is known and thus it corresponds morphologically and genetically with data presented herein and in Anastasiadou et al. (2006); (6) the neotype is selected from the “Maine et Loire” sample in Bouvier (1913), corresponding to the area mentioned in Millet (1831); and (7) the neotype has been selected from a sample already belonging to MNHN (Na480). Therefore, all conditions of Art. 75 are considered to be met and the selection of neotype is justified.

In light of the current revision of the species complex across Europe, North Africa and the Middle East, a nomenclatorial problem exists with the nomen, *Atyaephyra desmarestii* var. *occidentalis* Bouvier (1913), for which Bouvier (1913) did not designate a holotype. As such, the syntypic material of this variety (considered to be equivalent to a subspecies under Art. 45.6.4) includes all the material listed by Bouvier (1913) to have originated from North Africa and southern Europe, up to Macedonia. As such, this includes material from the Vardar region as summarily listed in Bouvier (1913), the area from which subsequently *Atyaephyra desmarestii stankoi* Karaman (1972) was described. As the name of Bouvier’s variety would take precedence over *Atyaephyra stankoi* as used in the present revision (a precedence which would cause considerable confusion), the herein selected neotype of *Atyaephyra desmarestii* (see above) is simultaneously selected as the lectotype of *Atyaephyra desmarestii* var. *occidentalis* Bouvier, 1913. This being fully justified by the inclusion of the “Maine et Loire” material in Bouvier (1913)’s type series. As a result of this action, the nomen *Atyaephyra stankoi* Karaman, 1972 can be used for the Macedonian taxon (as used herein), whilst *Atyaephyra desmarestii* var. *occidentalis* Bouvier, 1913 becomes a junior synonym of *Atyaephyra desmarestii* (Millet, 1831).

Bouvier (1913) also mentions he examined material from Coimbra (Portugal), with those particular specimens send by “*Barboza*” from the Museu Bocage under the name *Atyaëphyra rosiana*. He further indicates that these almost surely are cotypes from Brito Capello (“presque sûrement des cotypes”). These specimens are still present in the collection of MNHN (registration number Na509), with the label information (see Appendix: Fig. 4) corroborating the statement in Bouvier (1913) and as such are herein interpreted as syntypes of *Atyaephyra rosiana* de Brito Capello, 1867. Under ICZN Art. 75.8, the neotype selected by Anastasiadou et al. (2008) is thus set aside by the rediscovery of these syntypes. As the synonymy of *Atyaephyra rosiana* with *Atyaephyra desmarestii* seems certain at present, there appears currently no need to select a lectotype amongst the material. It should however be noted that the type locality of *Atyaephyra rosiana* de Brito Capello, 1867 reverts back to Coimbra (Portugal) and is no longer São Barnabe River, Algarve, as listed in De Grave & Fransen (2011) (see also García Muñoz et al. 2009).

**Figure 6.**
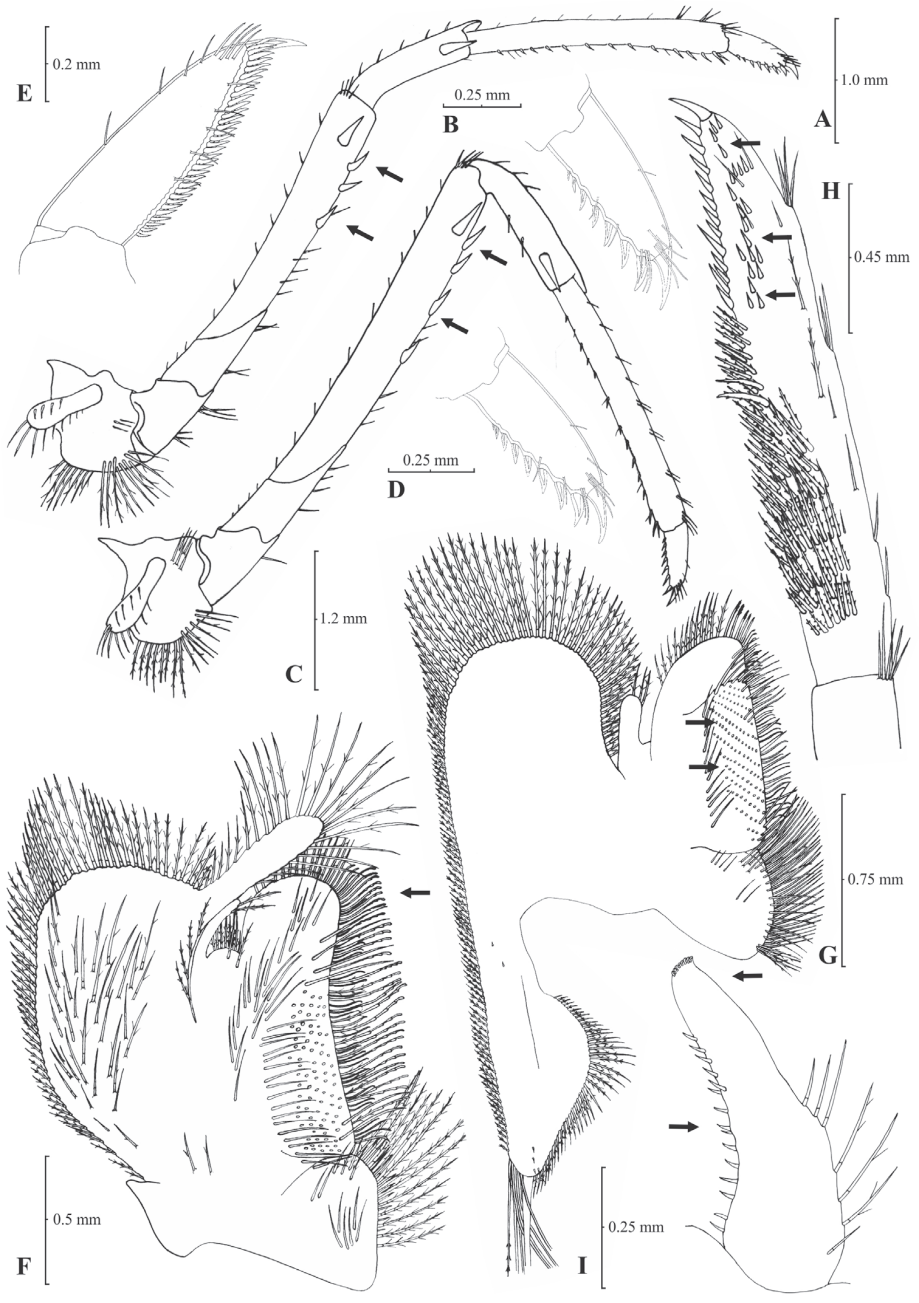
*Atyaephyra stankoi* Karaman, 1972. Neotype, adult ♀ (NHM 2012.1475): **A** rightthird pereiopod **B** dactylus of third pereiopod **C** rightfourth pereiopod **D** dactylus of fourth pereiopod **E** dactylus of fifth pereiopod **F** rightfirst maxilliped **G** rightmaxilla **H** right terminal segment of third maxilliped. Adult ♂ (ZMAUTH G1 988): **I** right endopod of first male pleopod.

*Atyaephyra desmarestii* can be distinguished among other characters from *Atyaephyra stankoi*, *Atyaephyra orientalis* and *Atyaephyra thyamisensis* sp. n. by the presence of 0–8 mesial spines (Anastasiadou et al. 2006; Fig. 3G) on the terminal segment of third maxilliped (vs. 10–38 in *Atyaephyra orientalis*, *Atyaephyra stankoi* and *Atyaephyra thyamisensis* sp. n.; Figs 4H, 6H, 8H respectively) and by the basal endite of first maxilliped reaching beyond distal end of exopod (Anastasiadou et al. 2006; Fig. 3D) (vs. basal endite fails to reach or reaches distal end of exopod in *Atyaephyra orientalis*, *Atyaephyra stankoi* and *Atyaephyra thyamisensis* sp. n.; Figs 4F, 6F, 8F respectively). *Atyaephyra desmarestii* is similar to *Atyaephyra strymonensis* sp. n. in having 0–8 mesial spines on the terminal segment of third maxilliped (Fig. 10H) but it can be discriminated by the presence of 1–5 post orbital rostral teeth (Anastasiadou et al. 2006; Fig. 1) (vs. no post orbital teeth present leaving short unarmed proximal gap in *Atyaephyra strymonensis* sp. n.; Fig. 9A).

#### Atyaephyra orientalis

Bouvier, 1913

http://species-id.net/wiki/Atyaephyra_orientalis

Figs 3 –4

Hemicaridina Desmaresti . – Barrois 1893: 126–134: Figs 1–3.
Atyaephyra desmarestii var. orientalis Bouvier, 1913: 65–74, Figs 1, 3C [type locality: Syria].
Atyaephyra desmaresti . – Annandale and Kemp 1913: 241–244.
Atyaëphyra Desmaresti . – Bouvier 1925: 84–89 Figs 159–162, partim.
Atyaephyra desmarestii orientalis . – Holthuis 1961: 5–10, Figs 2C–E, 3C–H; Kinzelbach and Koster 1985: 127–133, Fig. 1, partim.
Atyaephyra desmarestii mesopotamica Al-Adhub, 1987: 1–4, Fig. 1 [type locality: Shatt Al-Arab River and Hammar Lake, Iraq]. – Salman 1987: 27–42, Figs 1–8.
Atyaephyra desmarestii . –Gorgin 1996: 662–668, Figs 1–2; Anastasiadou et al. 2004: 5–13, partim; Von Rintelen et al. 2012: 82–96, partim.

##### Material examined.

**Turkey:** 3 ♀♀ (CL 4.8–5.0 mm), Antalya, Kirkgoz Spring (Fig. 1, stn 107), 21.6.2006, coll. M. Özbek; 7 ♀♀ (CL 4.5–5.5 mm), SMF 12174, Akbez, Karasu River (Fig. 1, stn 108), 22.9.1982, coll. R.K. Kinzelbach. **Syria:** 10 ♀♀ (3 ovig.) (CL 5.0–6.0 mm) and 4 ♂♂ (CL 4.0–5.0 mm), SMF 12050, below the dam of Ascharna, Orontes River (Fig. 1, stn 109), 30/31.3.1979, coll. R.K. Kinzelbach; 34 ♀♀ (15 ovig.) (CL 4.1–4.8 mm), SMF 12188, north of M’adan, Euphrates River (Fig. 1, stn 110), 17.8.1978, coll. R.K. Kinzelbach; 3 ♀♀ (2 ovig.) (CL 4.5–5.6 mm), SMF SYR8, Euphrates River (Fig. 1, stn 111), 15/16.6.1998, coll. R. Beck. **Israel:** 3 ♀♀ (2 ovig.) (CL 4.7–5.3 mm) and 2 ♂♂ (CL 3.9–4.0 mm), SMF IES 1189, Te’o Spring (Fig. 1, stn 112), 16.2.1977; 9 ♀♀ (CL 4.3–6.0 mm) and 4 ♂♂ (CL 3.9–4.0 mm), Hula Lake (Fig. 1, stn 113), 29.1.1981, coll. D. Eurth; 2 ovig. ♀♀ (CL 3.8–3.9 mm), NHM 1913.7.24.3–12, Kinneret Lake (Fig. 1, stn 114), 24.7.1913; 1 ♀ (CL 3.9 mm), Samakh, Kinneret Lake, 6.5.1986, coll. R. Ortal; 1 ♀ (CL 4.4 mm), Zaki River (Fig. 1, stn 115), 6.5.1986, coll. R. Ortal; 1 ♀ (CL 4.0 mm), Jordan River (Fig. 1, stn 116), 6.5.1981, coll. R. Ortal; 1 ♀ (CL 4.2 mm) and 1 ♂ (CL 3.8 mm), NHM 1938.1.26.8.12, Jordan River, 26.1.1938. **Jordan:** 2 ♀♀ (1 ovig.) (CL 4.0–4.9 mm), SMF 12057, Al-Azraq Oasis (Fig. 1, stn 117), 24.3.1977, coll. H. Damian. **Iraq:** 12 ♀♀ (CL 5.6–6.8 mm) and 3 ♂♂ (CL 4.5–4.8 mm), Basrah, Garmat Ali marsh (Fig. 1, stn 118), 24.2.1987, coll. A.H.Y. Al-Adhub; 1 ♀ (CL 5.2 mm), NHM 1919.11.14.5–20, Basrah, Shatt Al-Arab River (Robat creek) (Fig. 1, stn 119), 14.11.1919, coll. Capt. Boulenger; 1 ♂ (CL 4.2 mm), NHM 1919.4.28.2–3, Basrah, Shatt Al-Arab River (Robat creek), 28.4.1919, coll. P.J. Barraud; 4 ♀♀ (1 ovig.) (CL 5.2–5.5 mm) and 1 ♂ (CL 4.8 mm), Basrah, Shatt Al-Arab River (Fig. 1, stn 120), 2011, coll. M.D. Naser; 1 ovig. ♀, NHM 1919.11.12.11, Amarah, Tigris River (Fig. 1, stn 121), 12.11.1919, coll. J.O. Cooper Esq.

##### Amendments to description.

Rostrum long, slender, dorsal margin straight, slightly or strongly curved in the middle and pointed upwards or downwards, 6.0–10.0, most frequently (91% of the individuals examined) 6.5–9.25, × as long as high, shorter or equal to, or longer than scaphocerite (longer in 71% of the individuals examined). 14–29 (18–23 in 80% of the individuals) pre orbital teeth on dorsal margin of rostrum arranged to tip. 0–3, most often (85%) 1–3, post-orbital teeth. 3–13 teeth, mostly (96%) 4–10, arranged on ventral margin of rostrum (Fig. 3A). Carapace smooth with pterygostomial angle not protruding and rounded or bluntly produced (Figs 3B–C). Pleuron of fifth abdominal segment pointed ending in an acute or an obtuse posterior angle (Fig. 3D). Telsonwith 3–6, predominantly (93%) 4–5, pairs of dorsal spines arranged in curved fashion (Fig. 3E). Distal border of telson with 7–12, most often (91%) 8–10, spines (4–5 pairs) arranged in a fork-like or a fan-like way. Outermost pair of spines shortest, similar to dorsal spines, adjacent pair stronger terminating beyond, along with or before (beyond and along with in 64% of the individuals) the inner finely setulose pairs (Figs 3E–F). Basal segment of antennular peduncle with long stylocerite, with its tip failing to reach, reaching or overreaching the distal end of basal segment. Anterolateral lobe of basal segment short and pointed (Figs 3H–I). Distal segment of antennular peduncle with 0–3, most often (93%) 1–2, spines (Fig. 3G). Basal lower endite of maxilla densely covered with long simple setae arranged in 11–16 (12–15 in 93% of the individuals) oblique parallel rows. Endite of maxilla 1.75–2.20, mostly (93%) 1.81–2.07, × as long as basal lower endite (Fig. 4G). Basal endite of first maxilliped failing or reaching to distal end of exopod distal margin (Fig. 4F). Distal one-third of terminal segment of third maxilliped bearing 10–36 (14–31 in 84% of the individuals), mesial spines and one subdistal lateral spine near the base of larger terminal spine (Fig. 4H). Armature along flexor margin of dactylus of third and fourth pereiopod consisting of 6–11 (7–10 in 97% of the individuals) and 7–11 (8–10 in 89% of the individuals) spines (including terminal spine) respectively (Figs 4B, 4D). Merus of third and fourth pereiopod with 6–10 (7–9 in 85% of the individuals) and 5–9 (6–7 in 83% of the individuals) spines respectively (Figs 4A, 4C). Dactylus of fifth pereiopod with 33–55 (36–49 in 83% of the individuals) spines arranged in comb-like fashion on flexor margin (Fig. 4E). Endopod of first male pleopod expanded proximally with a distal portion stout and not tapering, often, with a, large protruding lobe in its outer subdistal part. Endopod with 13–38 spines arranged on a strongly curved inner margin and 5–8 setae arranged on outer margin (Fig. 4I, Bouvier et al. 1913: Fig. 1). 32–158 eggs of 0.5–0.75 × 0.35–0.5 mm in size.

##### Size.

*Atyaephyra orientalis* is a small-medium sized species of *Atyaephyra*, with maximum carapace length to be 4.8 mm in ♂♂, 6.8 mm in ♀♀ and 5.5 mm in ovig. ♀♀.

##### Molecular characters.

*Atyaephyra orientalis* can be differentiated from all other species of *Atyaephyra* by molecular characters, as demonstrated by the phylogenetic analysis of mtDNA COI sequences. Additionally, 5 haplotypes, each from a different location, found in *Atyaephyraorientalis* were not shared by any other species of the genus. It also differs from all the other species in the following nucleotide positions in the COI gene of *Atyaephyra desmarestii* specimen Dour1, position 273: guanine (G), position 276: guanine (G) and position 369: cytosine (C).

##### Distribution.

*Atyaephyra orientalis* is found in freshwater habitats of Middle East, from Turkey to Iraq (see material examined and Fig. 1).

##### Remarks.

Bouvier (1913) after examining the *Atyaephyra* material deposited in the MNHN collections he assigned it into two varieties (*Atyaephyra desmarestii var. orientalis* and *Atyaephyra desmarestii occidentalis*) based mainly on differences observed in the endopod of first male pleopod. *Atyaephyra desmarestii var. orientalis* was originally described from Syria (from Orontes River, near the Lake Qattinah (Lake Homs), from a stream in Kousseir (probably Qoussair) near Damascus and from Barada River, Ataibe, East of Damascus) and was elevated to subspecies level by Holthuis (1961). Apart from *Atyaephyra desmarestii orientalis*, a second subspecies, *Atyaephyra desmarestii mesopotamica*, was found to exist in the Middle East and was described by Al-Adhub (1987). Al-Adhub (1987) described the new subspecies based on the presence of a distinct subterminal process (vs. absent from *Atyaephyra desmarestii orientalis* and *Atyaephyra desmarestii desmarestii*) and the presence of 50 spines on dactylus of fifth pereiopod (vs. 40 in *Atyaephyra desmarestii orientalis* and *Atyaephyra desmarestii desmarestii*). Furthermore he noticed that the rostrum of *Atyaephyra desmarestii mesopotamica* resembles that of *Atyaephyra desmarestii desmarestii* from Greece but differs in having the distal ventral part always devoid of teeth. Indeed the individuals from Shatt Al-Arab River had the highest number of spines on dactylus of fifth pereiopod ranging from 41–55 but specimens from the River Orontes were also found with up to 47 spines (33–47). Additionally, male individuals having endopod with a distinct subterminal process were found again in River Orontes as well as in other Middle East Rivers. Gorgin (1996), after studying 150 males from two different localities in Iran found individuals with a distinct subterminal process and without inside the same population. Finally, specimens from Greece belonging to *Atyaephyra stankoi* (as the sample of Holthuis to which Al-Adhub refers to) were found to be also devoid of teeth in the distal part of the rostrum. Even in the illustration included in Holthuis (1961) work, the Greek specimen is devoid of teeth in the distal part of the ventral margin. Although the genetic distances within the *Atyaephyra orientalis* phylogroup were high (0.9%–10.2%) no firm conclusion could be drawn whether the hypothesis of multiple species is valid or not. Sequences from Orontes River (topotypical location of *Atyaephyra desmarestii orientalis*) and from Shatt Al-Arab River (topotypical location of *Atyaephyra desmarestii mesopotamica*) presented a noticeable mean genetic divergence (5.0%) but still not strong enough to support the hypothesis of different species. Detailed future studies on the morphological and genetic variability within the *Atyaephyra* distributed throughout the Middle East will help clarify the relationships between the populations in this region. However, only one species is currently considered to exist, *Atyaephyra orientalis*. Therefore, *Atyaephyra desmarestii mesopotamica* is here proposed as a synonym.

**Figure 7.**
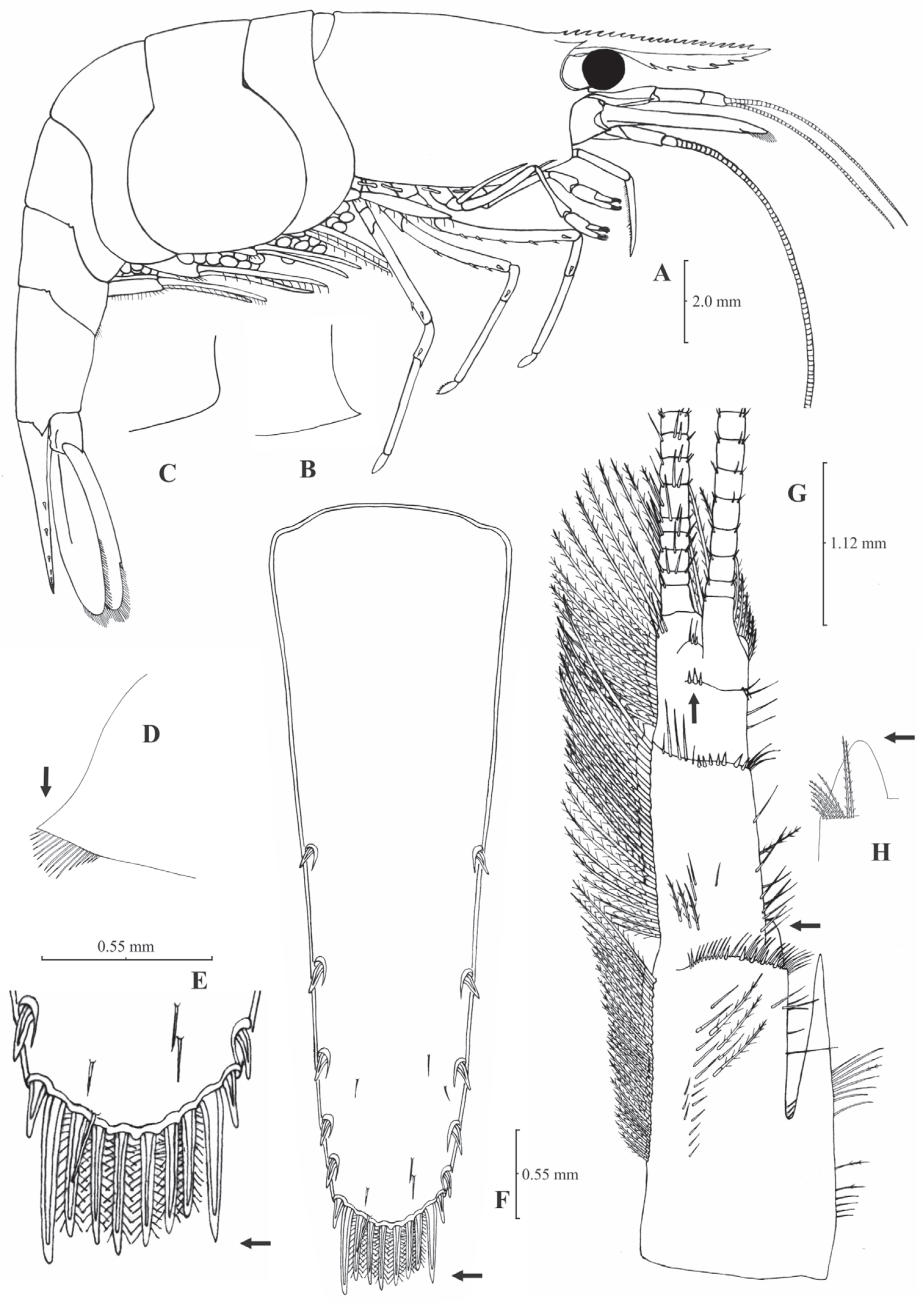
*Atyaephyra thyamisensis* sp. n. Holotype, adult ovig. ♀ (NHM 2012.1476): **A** entire individual **B** detail ofleftpterygostomial boarder **C** detail ofrightpterygostomial boarder **D** right pleuron of fifth abdominal segment **E** telson **F** distal margin of telson **G** right antennular peduncle **H** right antennular lobe.

**Figure 8.**
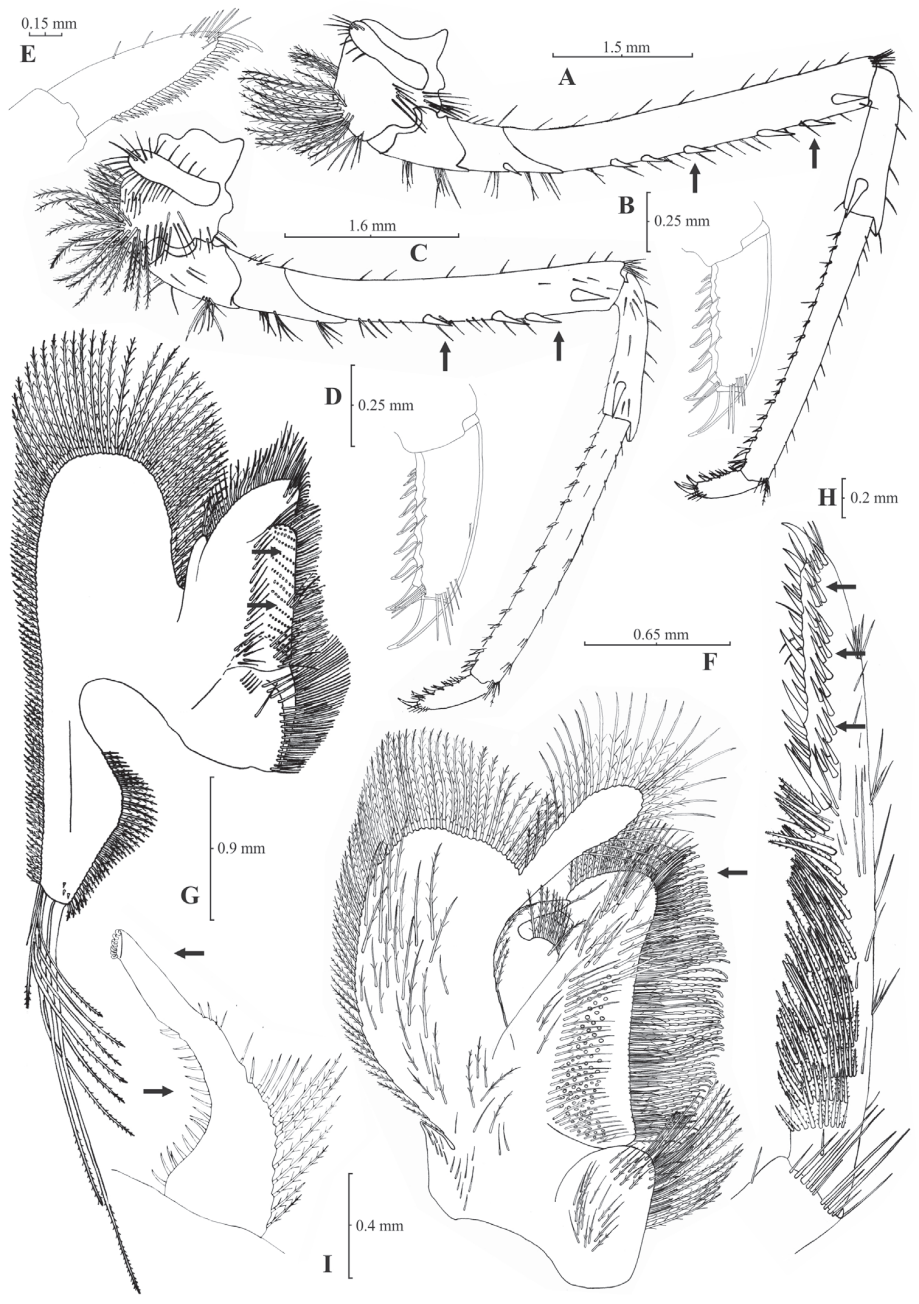
*Atyaephyra thyamisensis* sp. n. Holotype, adult ovig. ♀ (NHM 2012.1476): **A** right third pereiopod **B** dactylus of third pereiopod **C** rightfourth pereiopod **D** dactylus of fourth pereiopod **E** right dactylus of fifth pereiopod **F** rightfirst maxilliped **G** right maxilla **H** right terminal segment of third maxilliped. Allotype, adult ♂ (NHM 2012.1477): **I** rightendopod of first male pleopod.

*Atyaephyra orientalis* appears to be morphologically more similar to *Atyaephyra stankoi* and *Atyaephyra thyamisensis* sp. n. by sharing characters such as the presence of numerous mesial spines (10–38) on terminal segment of third maxilliped (Figs 4H, 6H, 8H). It also shares in common with the other two species the presence of fewer rows of setae (12–16) on basal lower endite of maxilla, the endite of maxilla being 1.75–2.24 × as long as basal lower endite (Figs 4G, 6G, 8G) and basal endite of first maxilliped failing or reaching to distal end of exopod distal margin (Figs 4F, 6F, 8F). *Atyaephyra orientalis* can be separated from *Atyaephyra thyamisensis* sp. n. and *Atyaephyra stankoi* by the presence of a pointed antennular lobe (Figs 3H–I) (vs. round in *Atyaephyra stankoi* and *Atyaephyra thyamisensis* sp. n. Figs 5H, 7H). Further, *Atyaephyra orientalis* can be distinguished by the strongly curved and distally stout and not tapering endopod of male first pleopod (Fig. 4I) (vs. slightly curved and distally more or less elongated but always tapering in *Atyaephyra stankoi*, Fig. 6I; slightly or strongly curved but always its distal part is elongated and tapering (ribbon shaped) in *Atyaephyra thyamisensis* sp. n., Fig. 8I). *Atyaephyra orientalis* differs from the other four species of *Atyaephyra* in having 10–36 spines on terminal segment of third maxilliped (Fig. 4H) (vs. 0–8 in *Atyaephyra desmarestii*, *Atyaephyra strymonensis* sp. n., *Atyaephyra acheronensis* sp. n. and *Atyaephyra tuerkayi* sp. n. Figs 10H, 12H, 14H).

#### Atyaephyra stankoi

Karaman, 1972

http://species-id.net/wiki/Atyaephyra_stankoi

Figs 5 –6

Atyaephyra Desmaresti var. occidentalis Bouvier, 1913: 65–74, Figs 2I, 3I, partim.
Atyaephyra desmarestii desmarestii . – Holthuis 1961: 5–10, Figs 2B, 3B, partim.
Atyaephyra desmarestii stankoi Karaman, 1972: 81–84, Figs 3, 6, 9, 10 [type locality: Doirani Lake, Greece].
Atyaephyra desmarestii . –Anastasiadou et al. 2004: 5–13, partim
Atyaephyra stankoi . – Garcia Muñoz et al. 2009: 32–42, partim
Atyaephyra sp. n. 3. – Christodoulou et al. 2010: partim

##### Material examined.

**Type material.** Neotype: NHM 2012.1475, adult ♀ (CL 6.0 mm), Greece–F.Y.R.O.M., Doirani Lake, (Fig. 1, stn 99), among aquatic plants, 9.11.1992, coll. S. Jovanovich and E. Stojkoska [here designated].

##### Non-type material.

**Greece:** 4 ♀♀ (CL 5.4–5.9 mm), Peloponnesus, Alfeios River (Fig. 1, stn 82), 24.9.2001, coll. Ch. Anastasiadou; 4 ♀♀ (CL 5.4–5.7 mm), Aitoloacarnania, Ozeros Lake (Fig. 1, stn 83), 22.11.2001, coll. Ch. Anastasiadou; 2 ovig. ♀♀ (CL 5.5–7.0 mm), Aitoloakarnania, Aitoliko, Acheloos River (Fig. 1, stn 84), 4.4.2002, coll. Ch. Anastasiadou; 3 ♀♀ (CL 5.0–5.5 mm), Aitoloakarnania, Trichonida Lake (Fig. 1, stn 85), 22.10.2001, coll. Ch. Anastasiadou; 4 ♀♀ (CL 5.1–6.5 mm) Aitoloacarnania, Lysimachia Lake (Fig. 1, stn 86), 22.11.2001, coll. Ch. Anastasiadou; 1 ♀ (CL 6.9 mm) and 2 ♂♂ (CL 5.1–5.3 mm), Thessalia, Tavropou Lake (Fig. 1, stn 87), 14.11.2001, coll. Ch. Anastasiadou; 17 ♀♀ (CL 6.0–8.0) and 2 ♂♂ (CL 5.0 mm), Thessalia, Enipeas River (Fig. 1, stn 88), 14.10.2001, coll. Ch. Anastasiadou; 3 ♀♀ (CL 6.5–7.6 mm) and 1 ♂ (CL 5.5 mm), ZMAUTH G1-910, Thessalia, Mati Tyrnavou Lake (Fig. 1, stn 89), 15.11.1977, coll. A. Koukouras; 1 ♀ (CL 6.8 mm) and 1 ♂ (CL 5.2 mm) Thessalia, Pineios River (Fig. 1, stn 90), 15.11.2001, coll. Ch. Anastasiadou; 1 ♀ (CL 7.0 mm), Thessalia, Lithaios River (Fig. 1, stn 91), 14.11.2001, coll. Ch. Anastasiadou; 5 ♀♀ (CL 6.0–7.0 mm) and 1 ♂ (CL 5.0 mm), Thessalia, Gritsas River (Fig. 1, stn 92), 15.11.2001, coll. Ch. Anastasiadou; 3 ♀♀ (CL 6.0–6.7 mm), Macedonia, Aliakmonas River (Fig. 1, stn 93), 9.9.1974 and 26.11.1978; 4 ♀♀ (2 ovig.) (CL 5.7–6.8 mm), ZMAUTH G1-1005, Macedonia, Vegoritida Lake (Fig. 1, stn 94), 17.6.1968; 4 ♀♀ (1 ovig.) (CL 5.5–6.3 mm), ZMAUTH G1-1018, Thessalia, Agra Lake (Fig. 1, stn 95), 17.6.1968, coll. P. Economides; 12 ♀♀ (CL 5.5–7.0 mm) and 3 ♂♂ (CL 5.0–5.5 mm), Thessalia, Edessaios River (Fig. 1, stn 96), 19.10.2001, coll. Ch. Anastasiadou; 5 ♀♀ (CL 5.0–5.5 mm) and 1 ♂ (CL 5.0 mm), Thessalia, Kariotissa, Moglenitsa River (Fig. 1, stn 97), 18.10.2001, coll. Ch. Anastasiadou; 4 ♀♀ (CL 6.0–7.0 mm) and 1 ♂ (CL 5.0 mm), ZMAUTH G1-988, Macedonia, Axios River (Fig. 1, stn 98), 16.7.1971, coll. P. Economides; 11 ♀♀ (CL 5.9–7.3 mm) and 1 ♂ (CL 5.1 mm), Macedonia, Richios River (Fig. 1, stn 100), 26.10.01, coll. Ch. Anastasiadou; **Greece**–**F.Y.R.O.M.:** 4 ♀♀ (CL 5.0–5.7 mm), Doirani Lake, (Fig. 1, stn 99), 9.11.1992, coll. S. Jovanovich and E. Stojkoska.

##### Description.

Rostrum long, slender, dorsal margin straight or slightly curved in the middle and pointed upwards, 6.12–8.67, mostly (83% of the examined individuals) 6.25 to 7.54, × as long as high, shorter, equal to, or longer than scaphocerite (longer in 76% of the individuals examined). From 17 to 28 (19–27 in 91% of the individuals) pre orbital teeth on dorsal margin of rostrum arranged up to tip. 0–3, predominantly (96%) 1–3, post-orbital teeth. 2–8, most often (96%) 2–6, teeth arranged on ventral margin of rostrum (Fig. 5A). Carapace smooth with pterygostomial angle not protruding, rounded (Fig. 5B). Pleuron of fifth abdominal segment usually pointed ending in an obtuse (ending in an acute angle in 11% of the individuals) posterior angle (Figs 5C–D). Telsonwith 3–6, most often (93%) 5–6, pairs of dorsal spines arranged in curved fashion (Fig. 5E). Distal border of telson with 6–11, mostly (87%) 8–10, spines (3–6 pairs), arranged in a fork-like pattern. Outermost pair of spines shortest, similar to dorsal spines, adjacent pair stronger terminating beyond (or along with) the inner finely setulose pairs (Figs 5E–F). Basal segment of antennular peduncle with long stylocerite, with its tip failing to reach, reaching or overreaching the distal end of basal segment. Anterolateral lobe of basal segment short and rounded (Fig. 5H). Distal segment of antennular peduncle with 1–4, mostly (93%) 1–3, spines (Fig. 5G). Basal lower endite of maxilla densely covered with long simple setae arranged in 12–16, (13–15 in 89% of the individuals), oblique parallel rows. Endite of maxilla 1.78–2.08, mostly (89%) 1.84–1.99, × as long as basal lower endite (Fig. 6G). Basal endite of first maxilliped failing or reaching to distal end of exopod (Fig. 6F). Distal one-third of terminal segment of third maxilliped bearing 11–35, frequently (85%) 16–28, mesial spines and one subdistal lateral spine near the base of larger terminal spine (Fig. 6H). Armature along flexor margin of dactylus of third and fourth pereiopod consisting of 7–11 (7–9 in 98% of the individuals) and 7–10 (7–9 in 98% of the individuals) spines (including terminal spine) respectively (Figs 6B, 6D). Merus of third and fourth pereiopod with 3–8 (4–6 in 83% of the individuals examined) and 2–6 (3–5 in 88% of the individuals) spines respectively (Figs 6A, 6C). Dactylus of fifth pereiopod with 26–47, most often (80%) 32–41, spines arranged in comb-like fashion on flexor margin (Fig. 6E). Endopod of first male pleopod expanded proximally and with a distal portion either elongated (ribbon shape) or more stout but always tapering. Endopod with 13–17 spines arranged on a slightly curved inner margin and 7–12 setae arranged on the outer margin (Fig. 6I). 96–195 eggs of 0.6–0.7 × 0.4 mm in size.

##### Size.

*Atyaephyra stankoi* is a large sized species with maximum carapace length of 5.50 mm in ♂♂, 7.60 mm in ♀♀ and 6.8 mm in ovig. ♀♀.

##### Molecular characters.

*Atyaephyra stankoi* can be distinguished from all other species of *Atyaephyra* by molecular characters, as shown by the phylogenetic analysis of mtDNA COI sequences, such as the two unique *Atyaephyra stankoi* haplotypes. Furthermore, it differs from all the other species in the following nucleotide positions in the COI gene of *Atyaephyra desmarestii* specimen Dour1, position 192: cytosine (C), position 282: adenine (A), position 320: cytosine (C), position 342: cytosine (C) and position 423: cytosine (C).

##### Distribution.

*Atyaephyra stankoi* is found in freshwater habitats in the mainland of West-central Greece and South F.Y.R.O.M. (see material examined and Fig. 1).

##### Remarks.

Bouvier (1913) assigned the material of MNHN originating from Portugal, France, Corsica, Macedonia, Tunisia, Algeria and Morocco to var. *occidentalis* while the material from Syria he assigned to var. *orientalis*. The material from Macedonia was collected from the region of Vardar (Axios) north of Thessaloniki, from the Lake of Amatovo (drained in the early twentieth century) near Kirdzalar (today called Adendron). The two varieties described by Bouvier were elevated in subspecies level by Holthuis (1961) and the var *occidentalis* was re-named to *Atyaephyra desmaresii desmarestii* since it contained the name-bearing type of the species. Few years later, Karaman (1972) described a new subspecies from Doirani Lake which is part of the Vardar (Axios) basin and named it *Atyaephyra desmarestii stankoi* ignoring the available name of Bouvier’s (*Atyaephyra desmarestii var. occidentalis*). However, after designating a neotype of *Atyaephyra desmarestii* from Bouvier’s material the nomen *Atyaephyra desmarestii var. occidentalis* becomes unavailable since it becomes a junior synonym of *Atyaephyra desmarestii* (see *Atyaephyra desmarestii* remarks) and thus the nomen *Atyaephyra stankoi* can be used for the Macedonian taxon (as used herein).

Efforts made to trace Karaman’s type material in the MMNH were unsuccessful. According to the director of the Museum, Dr Petkovski S. (pers. comm.), Karaman’s material is considered lost after a fire that took place in the Museum.

A neotype for *Atyaephyra stankoi* is proposed for reasons of taxonomic clarification and stability, as foreseen by Art. 75 (ICZN, 1999). The neotype will contribute to the stability of the taxonomic status of the species and avoid further confusion due to nomenclature (see also *Atyaephyra desmarestii* remarks). Furthermore, it incorporates novel characteristics that distinguish it from the remaining *Atyaephyra* species such as: having 11–35 mesial spines on terminal segment of third maxilliped, basal endite of first maxilliped failing or reaching to distal end of exopod, distal boarder of telson with spines arranged in a fork-like pattern, a rounded antennular lobe, a pterygostomial angle not protruding, and a slightly curved and distally more or less elongated but always tapering endopod of male first pleopod. The name-bearing types are considered lost while the neotype has been collected from Doirani Lake, the same locality from where Karaman (1972) collected *Atyaephyra desmarestii stankoi* type material and it will replace the lost type material.

*Atyaephyra stankoi* is similar to *Atyaephyra thyamisensis* sp. n. in having: 11–38 mesial spines on terminal segment of third maxilliped (Figs 6H, 8H), 12–16 rows of setae on basal lower endite of maxilla (Figs 6G, 8G), 3–6 pairs (mostly 4–5) of spines on distal boarder of telson with the second pair to be the strongest and terminating beyond (or along with) the other pairs arranged in a fork-like pattern (Figs 5E–F, 7E–F), a rounded antennular lobe (Figs 5H, 7H) and the basal endite of first maxilliped failing or reaching to distal end of exopod (Figs 6F, 8F). *Atyaephyra stankoi* differs from *Atyaephyra thyamisensis* sp. n. in not having a sharply protruding pterygostomial angle (Figs 5B, 7B). *Atyaephyra stankoi* can be distinguished from *Atyaephyra orientalis* by the presence of a rounded antennular lobe (Fig. 5H) (vs. pointed in *Atyaephyra orientalis*; Figs 3H–I). Further, *Atyaephyra stankoi* can be distinguished by the slightly curved and distally more or less elongated but always tapering endopod of male first pleopod (Fig. 6I) (vs. strongly curved and distally stout and not tapering in *Atyaephyra orientalis*; Fig. 4I). *Atyaephyra stankoi* can be separated from *Atyaephyra desmarestii*, *Atyaephyra strymonensis*, *Atyaephyra acheronensis* and *Atyaephyra tuerkayi* by the presence of numerous mesial spines (11–35) on terminal segment of third maxilliped (Fig. 6H) (vs 0–8 mesial spines; Figs 10H, 12H, 14H).

#### Atyaephyra thyamisensis sp. n.

urn:lsid:zoobank.org:act:E57CE407-D38C-4EF2-B4AC-C0B9BEE6EFB1

http://species-id.net/wiki/Atyaephyra_thyamisensis

Figs 7 –8

Atyaephyra desmarestii . –Anastasiadou et al. 2004: 5–13, partim; Anastasiadou et al. 2011: 41–54, Figs 1–6.
Atyaephyra sp. n. 1. – Christodoulou et al. 2008: Fig. 4B.
Atyaephyra sp. n. 3. – Christodoulou et al. 2010: Fig. 2, partim.

##### Material examined.

**Type material.** Holotype: NHM 2012.1476, adult ovig. ♀ (CL 7.1 mm), Greece, Epirus, Thyamis River, 39°32.26'N, 20°09.76'E (Fig. 1, stn 76), among aquatic plants, 19.3.2005, coll. Ch. Anastasiadou; Allotype: NHM 2012.1477, adult ♂ (CL 5.3 mm), same data collection as holotype; Paratypes: NHM 2012.1478–1483, 4 ♀♀ (3 ovig.) (CL 6.0–6.8 mm) and 2 ♂♂ (CL 5.0–5.3 mm) same data collection as holotype. NHM 2012.1484–1485, 2 ♀ (CL 6.5–7.4 mm), Greece, Epirus, Louros River, 39°03.14'N, 20°46.26'E (Fig. 1, stn 72), among aquatic plants, 25.3.2012, coll. Ch. Anastasiadou. OUMNH.ZC 2012-08-001, 4 ♀♀ (2 ovig.) (CL 6.0–7.8 mm) and 2 ♂ (CL 5.2 mm) same data collection as holotype. SMF 43022, 4 ♀♀ (2 ovig.) (CL 5.8–7.1 mm) and 2 ♂♂ (CL 5.0–5.2 mm) same data collection as holotype. NHMW 25453, 4 ♀♀ (2 ovig.) (CL 5.5–7.5 mm) and 1 ♂♂ (CL 5.0 mm) same data collection as holotype

##### Non-type material.

**Greece:** 2 ♀♀ (CL 5.2–5.5 mm), NHMW 462, Corfu Island (Fig. 1, stn 75), 1.9.1937, coll. Stephanides; 13 ♀♀ (1 ovig.) (CL 5.3–8.1 mm) and 8 ♂♂ (CL 5.2–6.2 mm), Epirus, Thyamis River (Fig. 1, stn 77), 20.5.2000 and 26.10.01, coll. Ch. Anastasiadou; 20 ♀♀ (15 ovig.) (CL 6.5–7.5 mm) and 3 ♂♂ (CL 5.0–5.7 mm), Epirus, Pamvotida Lake (Fig. 1, stn 78), 24.3.2006, coll. Ch. Anastasiadou; 20 ♀♀ (CL 5.0–7.0) and 8 ♂♂ (CL 5.0–5.5), Epirus, Ziros Lake (Fig. 1, stn 79), 28.10.2001, coll. Ch. Anastasiadou; 20 ♀♀ (CL 5.8–8.5 mm) and 4 ♂♂ (CL 5.2–6.4 mm), ZMAUTH D-334, Epirus, Filipiada, Louros River (Fig. 1, stn 80), 20.10.1977, coll. P. Economides; 15 ♀♀ (CL 5.5–8.0) and 6 ♂♂ (CL 5.0–6.0), Louros River (Fig. 1, stn 80), 28.10.2001, coll. Ch. Anastasiadou; 8 ovig. ♀♀ (CL 6.4–8.0 mm) and 6 ♂♂ (CL 5.3–6.2 mm), NHMW 465, Lefkada Island, Kaligoni, Vardas River (Fig. 1, stn 81), Aug.1929, coll. Beier; 3 ovig. ♀♀ (CL 7.3–8.0 mm) and 3 ♂♂ (CL 5.0–5.9 mm), NHMW 466, Lefkada Island, Kaligoni, Vardas River (Fig. 1, stn 81), 2.10.1932, coll. Beier.

##### Description.

Rostrum long, slender, dorsal margin straight or slightly curved in the middle and pointed upwards, shorter, equal to, or longer than scaphocerite, 6.0–9.50, most often (84% of the examined individuals) 6.33 to 8.76, × as long as high. 18–27 (18–24 in 91% of the individuals) pre orbital teeth on dorsal margin arranged up to tip of rostrum. 0–2, predominantly (84%) 1–2, post-orbital teeth. 4–10 teeth, most often (87%) 5–8, arranged on ventral margin of rostrum (Fig. 7A). Carapace smooth with pterygostomial angle bluntly produced (Fig. 7B). Pleuron of fifth abdominal segment pointed with an acute posterior angle (Fig. 7D). Telsonwith 5–8, mostly (97%) 5–7, pairs of dorsal spines arranged in curved fashion (Fig. 7E). Distal border of telson with 8–12, mostly (86%) 8–10, spines (4–6 pairs) arranged in fork-like pattern. Outermost pair of spines shortest, similar to dorsal spines, adjacent pair stronger terminating beyond (or along with) the finely setulose inner pairs (Figs 7E–F). Basal segment of antennular peduncle with long stylocerite, with its tip reaching or overreaching the distal end of basal segment. Anterolateral lobe of basal segment short and round (Fig. 7H). Distal segment of antennular peduncle with 1–6, frequently (92%) 2–4, spines (Fig. 7G). Basal lower endite of maxilla densely covered with long simple setae arranged in 12–16 (13–15 in 80% of the individuals), oblique parallel rows. Endite of maxilla 1.84–2.24, mostly (93%) 1.89–2.05, × as long as basal lower endite (Fig. 8G). Basal endite of first maxilliped failing or reaching to distal end of exopod (Fig. 8F). Distal third of terminal segment of third maxilliped bearing 13–38 (19–30 in 88% of the individuals) mesial spines and one subdistal lateral spine near the base of larger terminal spine (Fig. 8H). Armature along flexor margin of dactylus of third and fourth pereiopod consisting of 6–9 (7–9 in 97% of the individuals) and 6–10 (7–9 in 97% of the individuals) spines respectively (Figs 8B, 8D). Merus of third and fourth pereiopod with 3–7 (4–6 in 93% of the individuals) and 2–6 (4–5 in 96% of the individuals) spines respectively (Figs 8A, 8C). Dactylus of fifth pereiopod with 28–43, usually (82%) 32–40, spines arranged in comb-like fashion on flexor margin (Fig. 8E). Endopod of first male pleopod expanded proximally and with a distal portion elongated (ribbon shaped) and tapering. Endopod with 14–21 spines arranged on a slightly or strongly curved inner margin and 12–18 setae arranged on outer margin (Fig. 8I). 172–465 eggs of 0.60–0.7 × 0.40–0.45 mm in size.

##### Size.

*Atyaephyra thyamisensis* sp. n. is a large sized species with a maximum carapace length of 6.4 mm in ♂♂, 8.0 mm in ♀♀ and 8.1 mm in ovig. ♀♀.

##### Molecular characters.

*Atyaephyra thyamisensis* sp. n. is different from all the other species of *Atyaephyra* by molecular characters, as shown by the phylogenetic analysis of mtDNA COI sequences. The one haplotype found was unique in the genus. Furthermore, it differs from all the other species in the following nucleotide positions in the COI gene of *Atyaephyra desmarestii* specimen Dour1, position 172: cytosine (C), position 207: cytosine (C), position 249: guanine (G), position 258: cytosine (C), position 324: guanine (G), position 348: guanine (G) and position 387: cytosine (C).

##### Etymology:

*Atyaephyra thyamisensis* sp. n. is named after the Thyamis River, Greece, the type locality.

##### Distribution.

*Atyaephyra thyamisensis* sp. n. is found in fresh water habitats of North-west Greece as well as in the islands Corfu and Lefkada (see material examined and Fig. 1).

**Remarks:** *Atyaephyra thyamisensis* can be discriminated from *Atyaephyra stankoi* by the presence of a sharply protruding pterygostomial angle (Fig. 7B). It should be noted that this character has been observed to be missing from one side (either the left or the right) in some very large sized individuals (Fig. 7C). This character is shared by *Atyaephyra orientalis* (present in some populations) along with the presence of numerous spines (10–38) on terminal segment of third maxilliped (Figs 4H, 8H) and the presence of fewer rows of setae (12–16) on basal lower endite of maxilla (Figs 4G, 8G). The two species can be distinguished by the presence of a rounded antennular lobe in *Atyaephyra thyamisensis* (Figs 7G–H) (vs. pointed in *Atyaephyra orientalis*; Figs 3G–I). Further, *Atyaephyra thyamisensis* can be distinguished by the slightly or strongly curved endopod of first male pleopod having its distal part always elongated and tapering (ribbon shaped; Fig. 8I) (vs. strongly curved and distally stout and not tapering in *Atyaephyra orientalis*; Fig. 4I). *Atyaephyra thyamisensis* can be separated easily from the remaining three species of *Atyaephyra* by the presence of numerous mesial spines (13–38; Fig. 8H) on terminal segment of third maxilliped (vs. 0–8 mesial spinesin *Atyaephyra desmarestii*, *Atyaephyra strymonensis*, *Atyaephyra acheronensis* and *Atyaephyra tuerkayi*; Figs 10H, 12H, 14H).

#### Atyaephyra strymonensis sp. n.

urn:lsid:zoobank.org:act:A0C25BDC-4FB3-4C41-A507-5FA0BF6BCFC7

http://species-id.net/wiki/Atyaephyra_strymonensis

Figs 9 –10

Atyaephyra desmarestii . –Anastasiadou et al. 2004: 5–13, partim; Sket and Zaksek 2009: 786–818.
Atyaephyra sp. n. 3. – Christodoulou et al. 2008.
Atyaephyra sp. n. 4. – Christodoulou et al. 2010: Fig. 2.

##### Material examined.

**Type material.** Holotype: NHM 2012.1486, adult ovig. ♀ (CL 7.0 mm), Greece, Macedonia, Mylopotamos Springs (Strymonas River), 41°08.90'N, 24°04.29'E (Fig. 1, stn 102), among aquatic plants, 23.5.2011, coll. M. Christodoulou and M.S. Kitsos. Allotype: NHM 2012.1487, adult ♂ (CL 5.0 mm), same data collection as holotype. Paratypes: NHM 2012.1488–1492, 4 ♀♀ (CL 5.2–7.0 mm) and 1 ♂ (CL 5.0 mm) same data collection as holotype. OUMNH.ZC 2012-08-002 4 ♀♀ (1 ovig.) (CL 5.2–7.0 mm) and 1 ♂ (CL 5.0 mm) same data collection as holotype; SMF 43023 2 ♀♀ (CL 6.7–7.2 mm) and 1 ♂ (CL 5.0 mm) same data collection as holotype; NHMW 25454, 2 ♀♀ (CL 6.1–7.3 mm) same data collection as holotype.

##### Non-type material.

**Greece:** 3 ♀♀ (CL 5.4–6.0 mm) Macedonia, Strymonas River (Fig. 1, stn 101), 1.10.2001, coll. Ch. Anastasiadou; 20 ♀♀ (13 ovig.) (CL 6.3–7.9 mm), Macedonia, Mylopotamos Springs (Fig. 1, stn 102), 4.4.2001, coll. Ch. Anastasiadou; 9 ♀♀ (CL 5.5–7.1 mm) and 5 ♂♂ (CL 5.1–5.3 mm) Macedonia, Agias Varvaras Springs (Fig. 1, stn 103), 4.4.2001, coll. Ch. Anastasiadou; 11 ♀♀ (4 ovig.) (CL 6.0–7.4 mm) and 3 ♂♂ (CL 5.1–5.3 mm), Macedonia, Kefalariou Springs (Fig. 1, stn 104), 4.5.2001, coll. Ch. Anastasiadou; 2 ♀♀ (CL 6.3 mm) and 2 ♂♂ (CL 5.3–5.6 mm), Thrace, Paradeisos, Nestos River (Fig. 1, stn 105), ZMAUTH G1-1024, 6.7.1972, coll. P. Economides; 14 ♀♀ (CL 5.5–7.3 mm) and 6 ♂♂ (CL 5.1–5.5 mm) Thrace, Kyrnos, Nestos River (Fig. 1, stn 106), 30.9.2002, coll. Ch. Anastasiadou.

##### Description.

**Figure 9.**
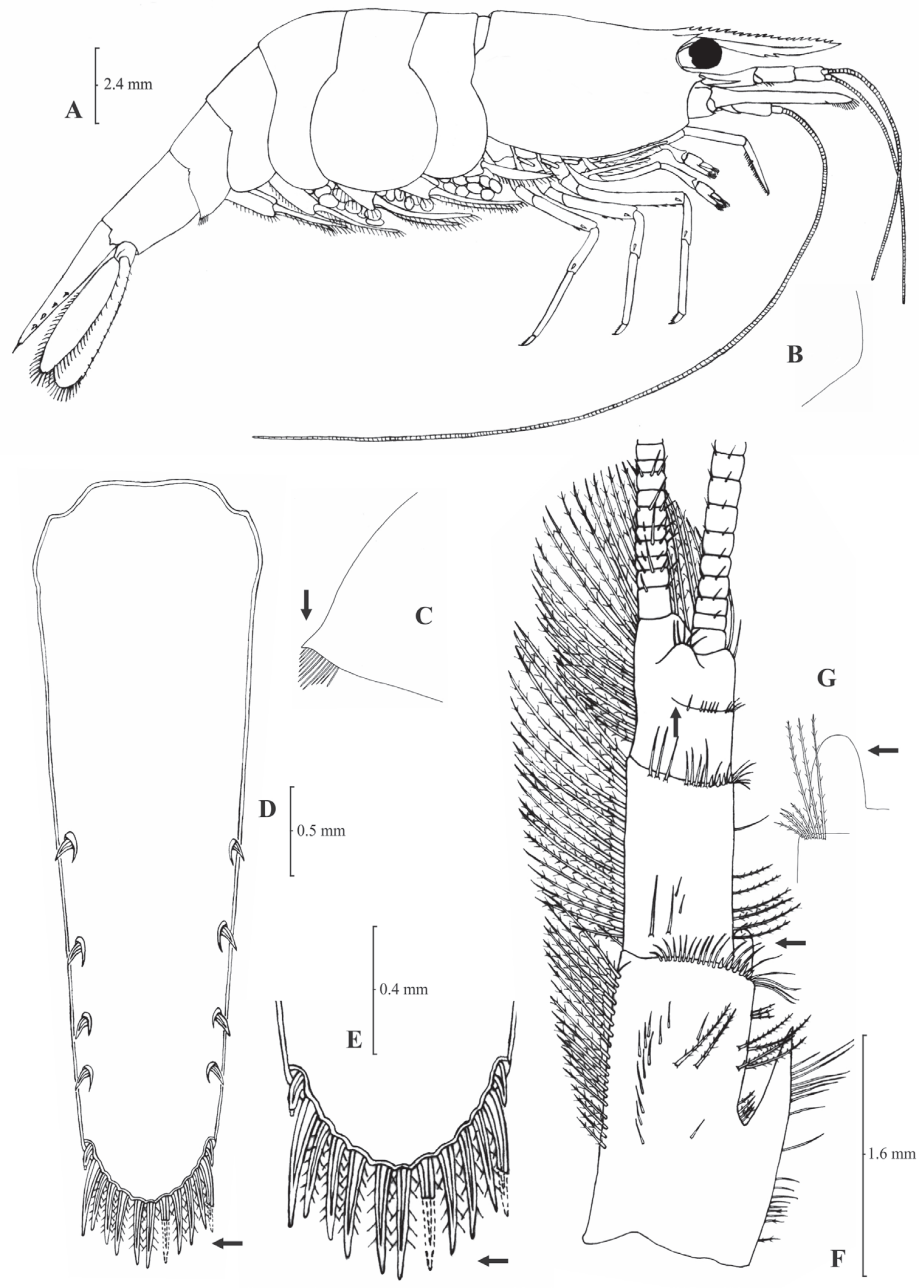
*Atyaephyra strymonensis* sp. n. Holotype, adult ovig. ♀ (NHM 2012.1486): **A** entire individual **B** detail of rightpterygostomial boarder **C** right pleuron of fifth abdominal segment **D** telson **E** distal margin of telson **F** right antennular peduncle **G** right antennular lobe.

**Figure 10.**
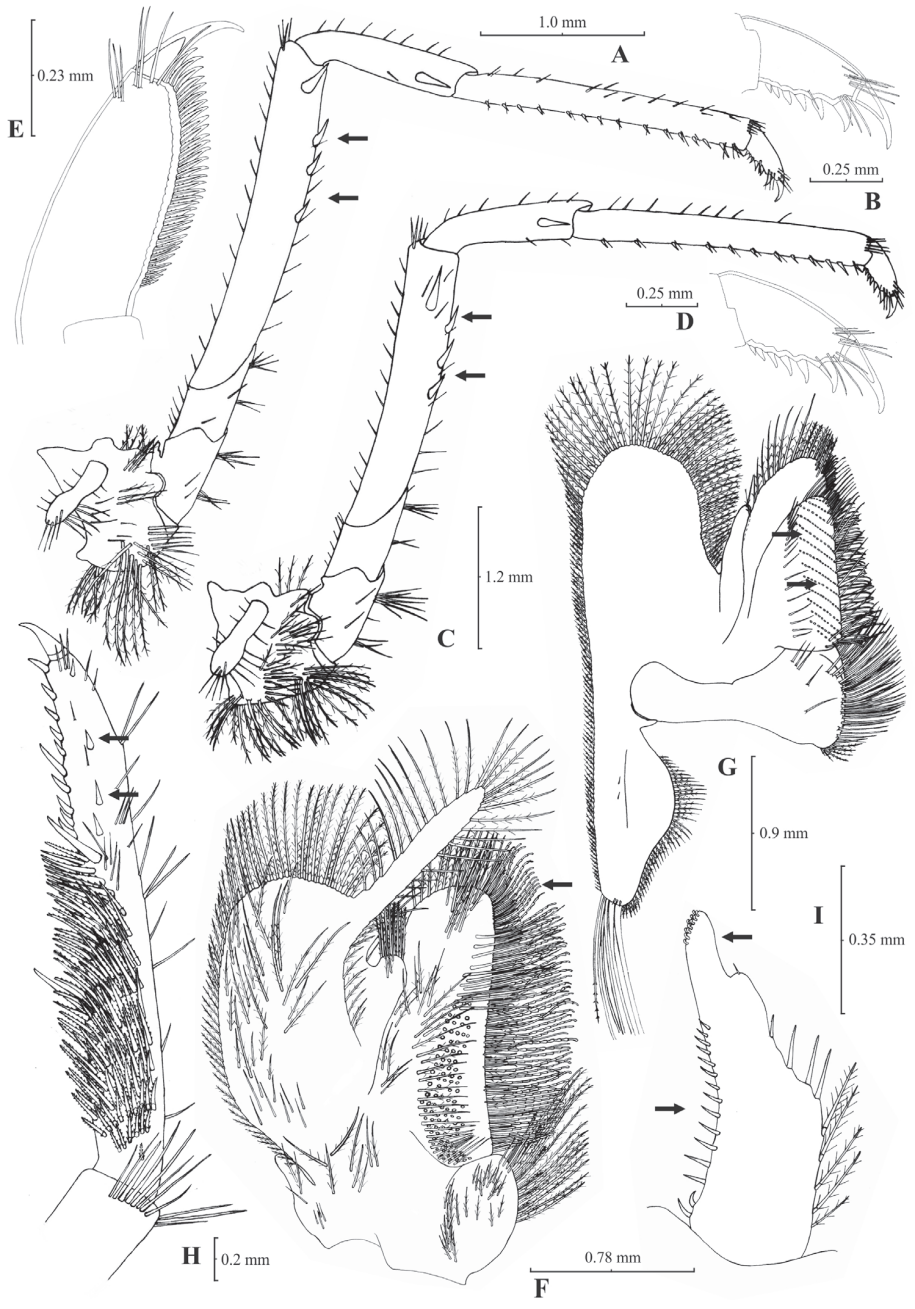
*Atyaephyra strymonensis* sp. n. Holotype, adult ovig. ♀ (NHM 2012.1486): **A** rightthird pereiopod **B** dactylus of third pereiopod **C** right fourth pereiopod **D** dactylus of fourth pereiopod **E** dactylus of fifth pereiopod **F** rightfirst maxilliped **G** right maxilla **H** rightterminal segment of third maxilliped. Allotype, adult ♂ (NHM 2012.1487): **I** right endopod of first male pleopod.

Rostrum long, slender, dorsal margin straight or slightly curved in the middle and pointed upwards, 5.89–8.80, mostly (92% of the individuals examined) 6.75–8.80, × as long as high, shorter, equal to, or longer than scaphocerite. 10–29, frequently (92%) 14–23, pre orbital teeth on dorsal margin of rostrum arranged up to tip. Rostrum without post-orbital teeth, leaving a short unarmed proximal gap. With maximally five teeth, mostly (91%) up to three, arranged on ventral margin of rostrum (Fig. 9A). Carapace smooth with pterygostomial angle, not protruding, rounded (Fig. 9B). Pleuron of fifth abdominal segment pointed with an acute posterior angle (Fig. 9C). Telsonwith 2–7, predominantly (97%) 3–4, pairs of dorsal spines arranged in curved fashion (Fig. 9D). Distal border of telson with 11–15, usually (96%) 12–14, spines (6–8 pairs), arranged in fan-like way. Outermost pair of spines shortest, similar to dorsal spines, adjacent pair stronger terminating before the finely setulose inner pairs (Figs 9D–E). Basal segment of antennular peduncle with long stylocerite, with its tip failing to reach or reaching the distal end of basal segment. Anterolateral lobe of basal segment short and round (Fig. 9G). Distal segment of antennular peduncle with 0–1 but mostly (87%) with no spines (Fig. 9F). Basal lower endite of maxilla densely covered with long simple setae arranged in 12–17 (14–16 in 90% of the individuals), oblique parallel rows. Endite of maxilla 1.77–1.95, mostly (89%) 1.78–1.91, × as long as basal lower endite (Fig. 10G). Basal endite of first maxilliped failing, reaching or overreaching the distal end of exopod (reaching the end in 65% of the individuals) (Fig. 10F). Distal one-third of terminal segment of third maxilliped bearing 1–7 mesial spines and one subdistal lateral spine near the base of larger terminal spine (Fig. 10H). Armature along flexor margin of dactylus of third and fourth pereiopod consisting of 6–8 (7–8 in 96% of the individuals) and 7–8 spines (including terminal spine) respectively (Figs 10B, 10D). Merus of third and fourth pereiopod with 3–6 (3–5 in 90% of the individuals) and 3–5 spines respectively (Figs 10A, 10C). Dactylus of fifth pereiopod with 25–37, mostly (87%) 30–35, spines arranged in comb-like fashion on flexor margin (Fig. 10E). Endopod of first male pleopod expanded proximally and with a distal portion elongated and tapering, often, with a small, protruding lobe in its outer subdistal part. Endopod with 14–23 spines arranged on a slightly curved inner margin and 9–15 setae arranged on outer margin (Fig. 10I). 210–250 eggs of 0.50–0.70 × 0.40–0.50 mm in size.

##### Size.

*Atyaephyra strymonensis* sp. n. is a large sized species with maximum carapace length to be 5.6 mm in ♂♂, 7.9 mm in ♀♀ and 7.5 mm in ovig. ♀♀.

##### Molecular characters.

*Atyaephyra strymonensis* sp. n. is unique in the genus in having 2 haplotypes not found in any of the other species. Also, it differs from all the other species in the following nucleotide positions in the COI gene of *Atyaephyra desmarestii* specimen Dour1, position 201: cytosine (C), position 252: guanine (G), position 303: cytosine (C), position 309: thymine (T), position 318: guanine (G), position 319: adenine (A), position 367: thymine (T), position 393: cytosine (C) and position 453: thymine (T).

##### Etymology:

*Atyaephyra strymonensis* sp. n. is named after the Strymon (Strymonas) River, Greece, the type locality.

##### Distribution.

*Atyaephyra strymonensis* sp. n. is found in North-western Greece in the Rivers Strymon and Nestos (see material examined and Fig. 1).

##### Remarks.

*Atyaephyra strymonensis* sp. n. is unique in the combination of the following characters: (a) absence of post orbital teeth (Fig. 9A), (b) leaving a short unarmed proximal gap on dorsal surface of rostrum (Fig. 9A), (b) having a round anterolateral lobe on basal segment of antennular peduncle (Figs 9F–G), (c) having a not protruding, rounded pterygostomial angle (Fig. 9C), (d) endite of maxilla 1.77–1.95 × as long as basal lower endite (Fig. 10G) and having 1–7 mesial spines in the terminal segment of third maxilliped (Fig. 10H). *Atyaephyra strymonensis* is similar to *Atyaephyra desmarestii*, *Atyaephyra acheronensis* and *Atyaephyra tuerkayi* in having fewer spines in the terminal segment of third maxilliped. However *Atyaephyra strymonensis* differs by the absence of post-orbital teeth, leaving a short unarmed proximal gap on dorsal surface of rostrum and by the endite of maxilla being 1.77–1.95 × as long as basal lower endite (vs. 1.49–1.71). *Atyaephyra strymonensis* differs from *Atyaephyra stankoi*, *Atyaephyra thyamisensis* and *Atyaephyra orientalis* in having fewer mesial spines in the terminal segment of third maxilliped.

#### Atyaephyra acheronensis sp. n.

urn:lsid:zoobank.org:act:EBF698A2-82F9-49E8-89DA-8C4EB7588939

http://species-id.net/wiki/Atyaephyra_acheronensis

Figs 11 –12

Atyaephyra sp. n. 2. – Christodoulou et al. 2008: Fig. 4A.
Atyaephyra sp. n. 2. – Christodoulou et al. 2010: Fig. 2, partim.
Atyaephyra desmarestii . – Franjević et al. 2010: 159–166.

##### Material examined.

**Type material.** Holotype: NHM 2012.1493, 1 ovig. ♀ (CL 5.9 mm), Greece, Epirus, Acherontas River, 39°13.96'N, 20°29.11'E (Fig. 1, stn 71), among aquatic plants, 15.4.2012, coll. Ch. Anastasiadou (Sequenced specimen: Ach1).

##### Non-type material.

**Greece:** 1 ♀ (CL 7.6 mm) (Sequenced specimen: Lour1) and 1 ovig. ♀ (CL 7.0 mm) (Sequenced specimen: Lour2), Greece, Epirus, Louros River, 39°03.14'N, 20°46.26'E (Fig. 1, stn 72), 15.4.2012, coll. Ch. Anastasiadou; **Slovenia:** 1 ♂ (CL 5.1 mm), Dragonja River (Fig. 1, stn 66), Aug.1971 (Sequenced specimen: Drag1).

##### Description.

**Figure 11.**
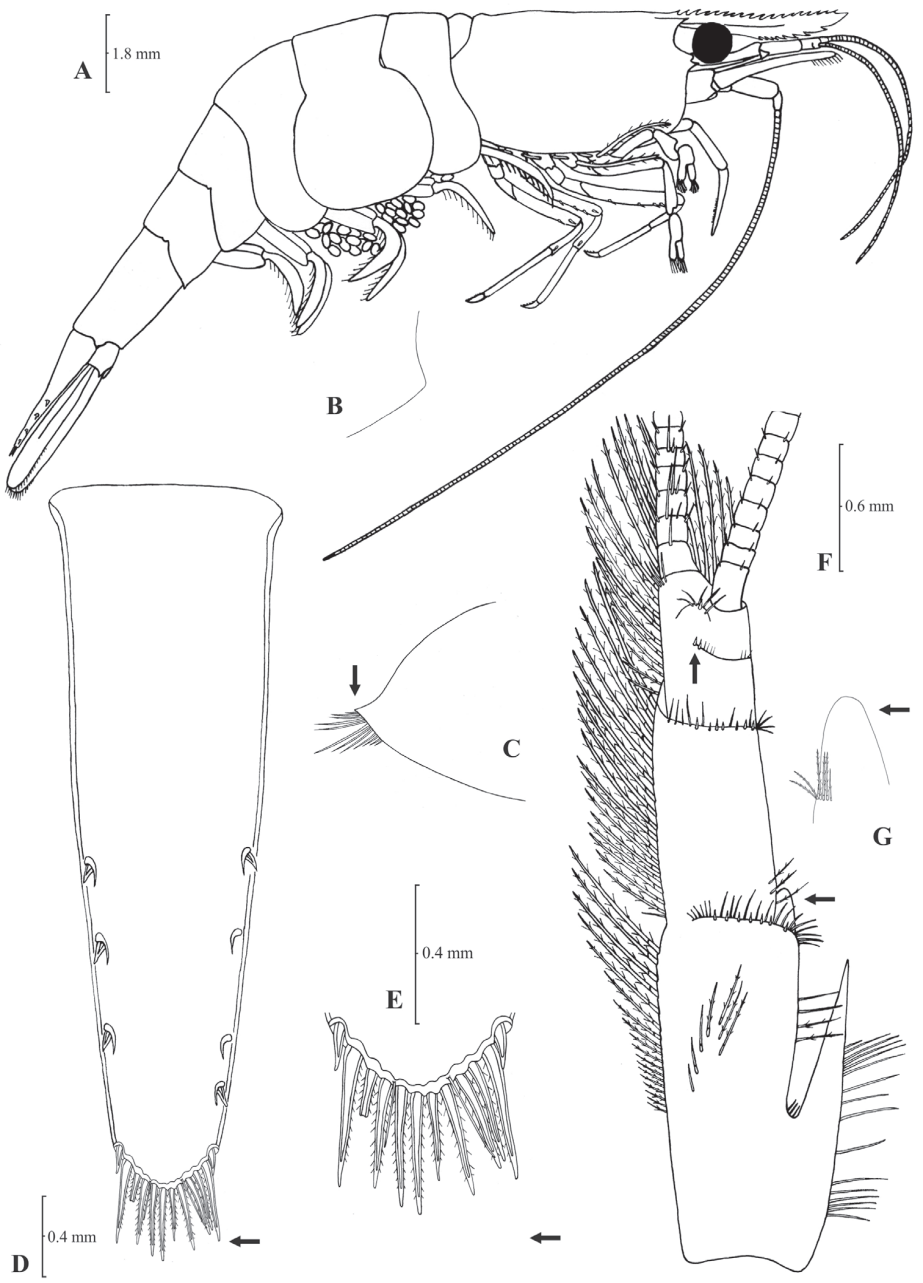
*Atyaephyra acheronensis* sp. n. Holotype, adult ovig. ♀ (NHM 2012.1493): **A** entire individual **B** detail of rightpterygostomial boarder **C** right pleuron of fifth abdominal segment **D** telson **E **distal margin of telson **F** right antennular peduncle **G** right antennular lobe.

**Figure 12.**
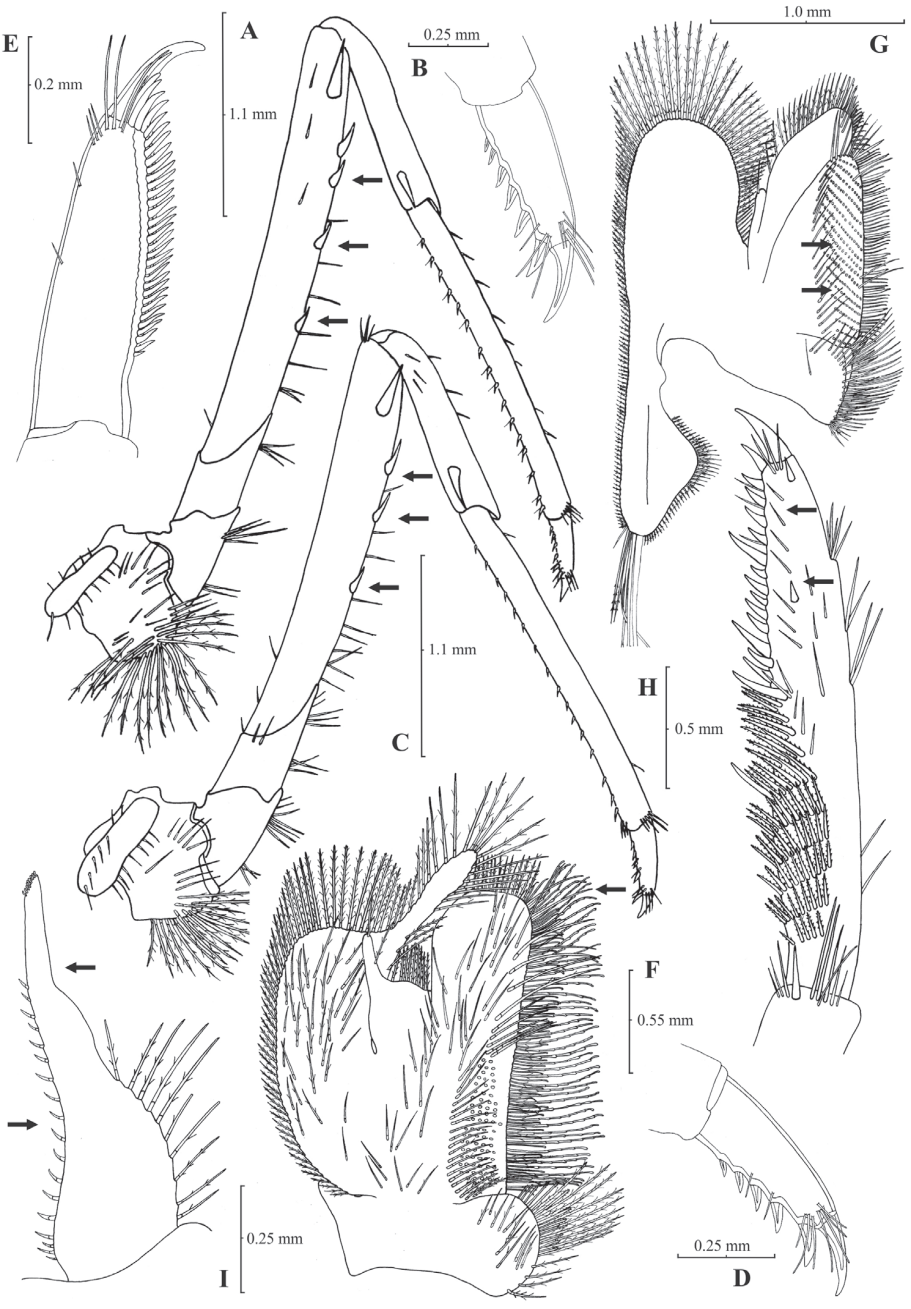
*Atyaephyra acheronensis* sp. n. Holotype, adult ovig. ♀ (NHM 2012.1493): **A** rightthird pereiopod **B** dactylus of third pereiopod **C** right fourth pereiopod **D** dactylus of fourth pereiopod **E**  dactylus of fifth pereiopod **F** rightfirst maxilliped **G** right maxilla **H** rightterminal segment of third maxilliped. Adult ♂: **I** right endopod of first male pleopod.

Rostrum long, dorsal margin straight, 6.28–6.66 × as long as high, equal to or longer than scaphocerite. 19–26 pre orbital teeth on dorsal margin of rostrum arranged up to tip. With 1–3 post orbital teeth and 3–8 teeth on ventral margin of rostrum (Fig. 11A). Carapace smooth with pterygostomial angle not protruding, rounded (Fig. 11B). Pleuron of fifth abdominal segment pointed with an acute posterior angle (Fig. 11C). Telsonwith four pairs of dorsal spines arranged in curved fashion (Fig. 11D). Distal border of telson with 12–15 spines (6–8 pairs) arranged in a fan-like pattern. Outermost pair of spines shortest, similar to dorsal spines, adjacent pair stronger terminating before the finely setulose, inner pairs (Figs 11D–E). Antennulary stylocerite with its tip failing to reach or reaching distal margin of basal peduncle segment. Anterolateral lobe of basal segment short and round (Fig. 11G). Distal segment of antennular peduncle with 1–2 spines (Fig. 11F). Basal lower endite of maxilla densely covered with long simple setae arranged in 18–20 oblique parallel rows. Endite of maxilla 1.56–1.65 × as long as basal lower endite (Fig. 12G). Basal endite of first maxilliped reaching clearly beyond distal end of exopod (Fig. 12F). Distal one-third of terminal segment of third maxilliped bearing 1–5 mesial spines and one subdistal lateral spine near the base of larger terminal spine, interpretable as dactylus (Fig. 12H). Armature along flexor margin of dactylus of third and fourth pereiopod consisting of 5–7 and 6–7 spines respectively (Figs 12B, 12D). Merus of third and fourth pereiopod with 4–6 and 3–4 spines respectively (Figs 12A, 12C). Armature along flexor margin of dactylus of fifth pereiopod consisting of 27–38 spines (Fig. 12E). Endopod of first male pleopod expanded proximally and with a distal portion elongated (ribbon shaped) and tapering. Endopod with 18 spines arranged on a slightly curved inner margin and 12 setae arranged on outer margin (Fig. 12I). 579–1117 eggs of 0.40–0.55 × 0.25–0.35 mm in size.

##### Size.

*Atyaephyra acheronensis* sp. n. is a large sized species with maximum carapace length to be 5.1 mm in ♂♂, 7.6 mm in ♀♀ and 7.0 mm in ovig. ♀♀.

##### Molecular characters.

Molecular information based on the COI sequences provides compelling evidence that is a well defined species.*Atyaephyra acheronensis* sp. n. is unique in *Atyaephyra* in having 2 haplotypes not shared by any other species. Furthermore, it differs from all its congeners in the following nucleotide positions in the COI gene of *Atyaephyra desmarestii* specimen Dour1, position 255: adenine (A) and position 318: cytosine (C). Finally, the mean genetic distances between *Atyaephyra acheronensis* and the remaining *Atyaephyra* species range from 8.3% to 23.8% (Table 2).

##### Etymology.

*Atyaephyra acheronensis* sp. n.is named after the Acheron (Acherontas) River, Greece, the type locality.

##### Distribution.

*Atyaephyra acheronensis* sp. n. is found in freshwater habitats of Croatia (Krka River), Slovenia (Dragonja River) and Greece (Acherontas River and Louros River) (see material examined and Fig. 1). Although this study was based on a limited number of specimens, it is postulated that *Atyaephyra acheronensis* sp. n. occurs in more rivers covering an area ranging from Croatia to Greece.

##### Remarks.

In addition to the type- and non type-material we investigated the morphology of the following specimens originating from the Balkan Peninsula: 6 ♀♀ collected from Dragonja River (Fig. 1, stn 66), Slovenia; 3 ♀♀ collected from Jadro River (Fig. 1, stn 67), NHMW 460 and 4 ♀♀ (3 ovig.) and 1 ♂ from Ombla River (Fig. 1, stn 69), NHMW 459, Croatia; 2 ♂♂ collected from Krupa River (Fig. 1, stn 68), NHMW 458, Bosnia and Herzegovina; 9 ♀♀ and 12 ♂♂ from Aoos River (Fig. 1, stn 70), Albania; 47 ♀♀ (13 ovig.) and 9 ♂♂ from Acherontas River (Fig. 1, stn 71), Greece, 10 ♀♀ and 2 ♂♂ collected from Louros River (Fig. 1, stn 72), Greece, 2 ♀♀ from Pamisos River (Fig. 1, stn 73), Greece, 4 ♀♀ and 1 ♂ sampled from Evrotas River (Fig. 1, stn 74), NHM 1987.93, Greece. However, without sequencing the individuals, their placement to *Atyaephyra acheronensis* sp. n. can’t be made with certainty.

Out of the 135 characters examined (see Appendix: Table 1) there were no morphological features distinguishing *Atyaephyra acheronensis* sp. n. from *Atyaephyra desmarestii* and *Atyaephyra tuerkayi* sp. n. Nevertheless, *Atyaephyra acheronensis* sp. n. presents a more limited variability in the values of its morphological characters than *Atyaephyra desmarestii*. *Atyaephyra acheronensis* sp. n. can easily be distinguished from *Atyaephyra orientalis*, *Atyaephyra stankoi* and *Atyaephyra thyamisensis* by the presence of fewer mesial spines (1–5) on terminal segment of third maxilliped (Fig. 12H) (vs. 10–38 in *Atyaephyra orientalis*, *Atyaephyra stankoi* and *Atyaephyra thyamisensis*; Figs 4H, 6H, 8H) and by the basal endite of first maxilliped overeaching distal end of exopod (Fig. 12F) (vs. failing to reach or reaching distal end in *Atyaephyra orientalis*, *Atyaephyra stankoi* and *Atyaephyra thyamisensis*; Figs 4F, 6F, 8F). *Atyaephyra acheronensis* sp. n. can be separated from *Atyaephyra strymonensis* by the presence of 1–3 post orbital rostral teeth (Fig. 11A) (vs. no post orbital teeth present leaving short unarmed proximal gap in *Atyaephyra strymonensis*; Fig. 9A) and by the endite of maxilla being 1.56–1.65 × as long as basal lower endite (Fig. 12G) (vs. 1.77–1.95 in *Atyaephyra strymonensis*; Fig. 10G).

#### Atyaephyra tuerkayi sp. n.

urn:lsid:zoobank.org:act:94C1EC2A-1667-4456-8721-D10F03CDF4E6

http://species-id.net/wiki/Atyaephyra_tuerkayi

Figs 13 –14

Atyaephyra desmarestii orientalis . – Kinzelbach and Koster 1985: 127–134, partim.
Atyaephyra n. sp. 2. – Christodoulou et al. 2010: Fig. 2, partim.

##### Material examined.

**Type material.** Holotype: adult ♀ (CL 6.2 mm), SMF 43020, Syria, Nahr Al-Kabir River (Fig. 1, stn 122), at bridge near the coastal road, 5.3.1979, coll. R.K. Kinzelbach (Sequenced specimen: Nah1); Paratype: 1 ♀ (CL 7.1 mm), SMF 43021 same data as the holotype (Sequenced specimen: Nah2).

##### Description.

**Figure 13.**
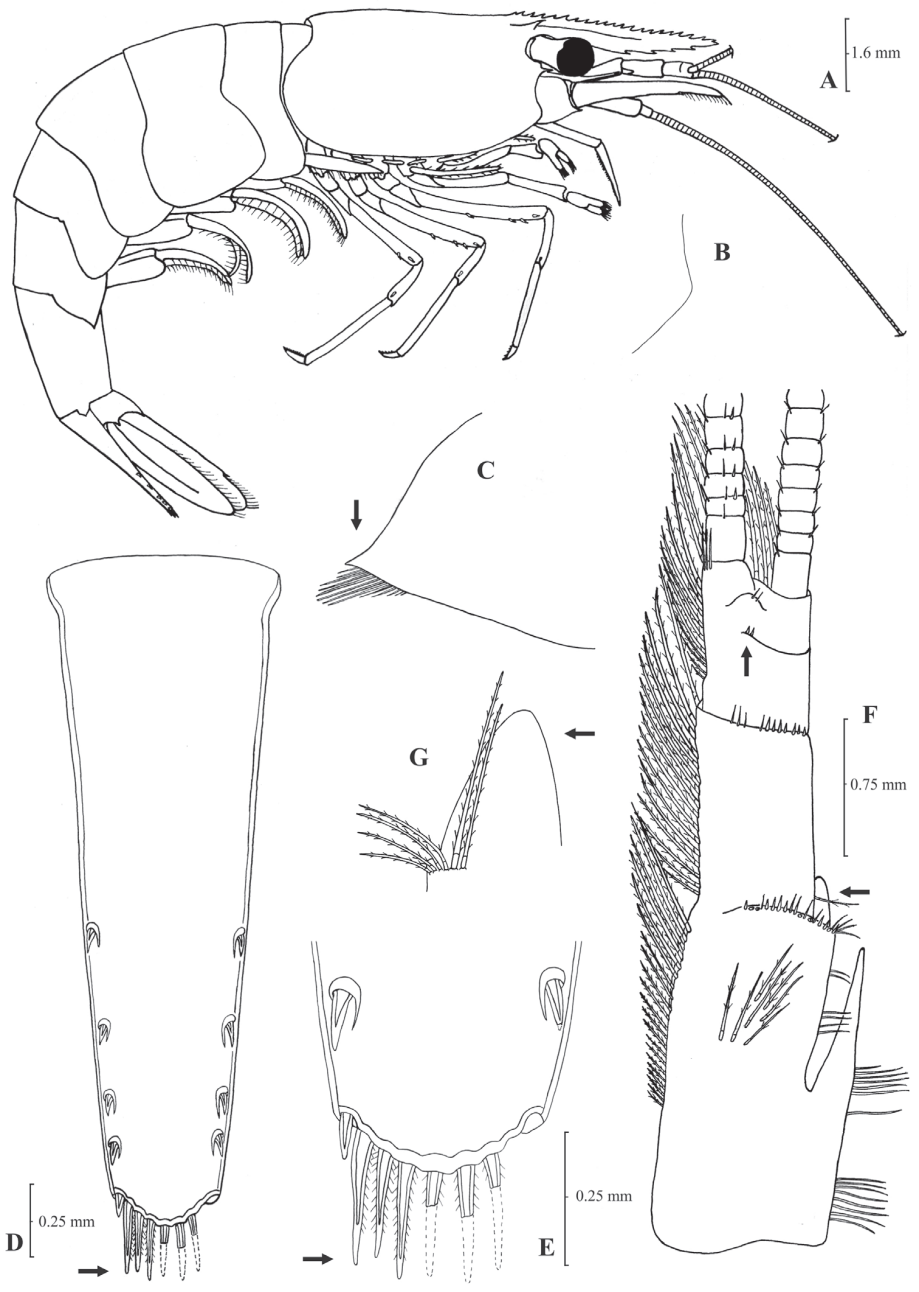
*Atyaephyra tuerkayi* sp. n. Holotype, adult ♀ (SMF 43020): **A** entire individual **B** detail of rightpterygostomial boarder **C** right pleuron of fifth abdominal segment **D** telson **E** distal margin of telson **F** right antennular peduncle **G** right antennular lobe.

**Figure 14.**
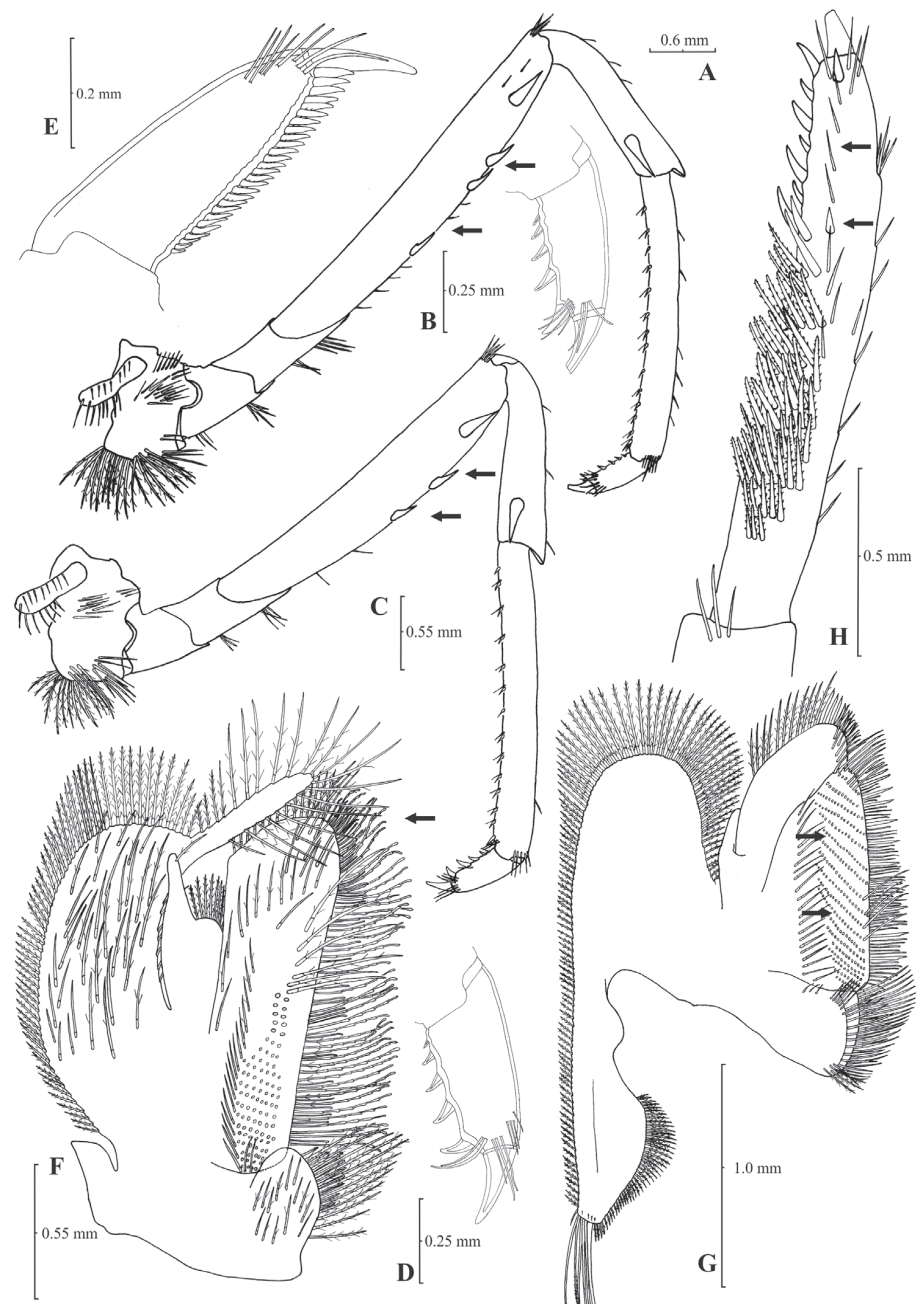
*Atyaephyra tuerkayi* sp. n. Holotype, adult ♀ (SMF 43020): **A** rightthird pereiopod **B **dactylus of third pereiopod **C** right fourth pereiopod **D** dactylus of fourth pereiopod **E** dactylus of fifth pereiopod **F** rightfirst maxilliped **G** right maxilla **H** rightterminal segment of third maxilliped.

Rostrum long, dorsal margin slightly curved in the middle and pointed upwards 6.43–6.66 × as long as high, shorter than or equal to scaphocerite. 19–23 pre orbital teeth on dorsal margin of rostrum arranged up to tip. With two post orbital teeth and 4–7 teeth on ventral margin of rostrum (Fig. 13A). Carapace smooth with pterygostomial angle not protruding, rounded (Fig. 13B). Pleuron of fifth abdominal segment pointed with an acute posterior angle (Fig. 13C). Telsonwith four pairs of dorsal spines arranged in curved fashion (Fig. 13D). Distal border of telson with 9 spines (5 pairs) arranged in fan-like pattern. Outermost pair of spines shortest, similar to dorsal spines, adjacent pair stronger terminating before the finely setulose, inner pairs (Fig. 13E). Antennulary stylocerite with its tip failing to reach or reaching distal margin of basal peduncle segment. Anterolateral lobe of basal segment short and round (Fig. 13G). Distal segment of antennular peduncle with 1–2 spines (Fig. 13F). Basal lower endite of maxilla densely covered with long simple setae arranged in 18–20 oblique parallel rows. Endite of maxilla 1.58–1.59 × as long as basal lower endite (Fig. 14G). Basal endite of first maxilliped reaching clearly beyond distal end of exopod (Fig. 14F). Distal one-third of terminal segment of third maxilliped bearing 1–6 mesial spines and one subdistal lateral spine near the base of larger terminal spine (Fig. 14H). Armature along flexor margin of dactylus of third and fourth pereiopod consisting of 6–7 and 6–7 spines respectively (Figs 14B, 14D). Merus of third and fourth pereiopod with 4 and 3 spines respectively (Figs 14A, 14D). Armature along flexor margin of dactylus of fifth pereiopod consisting of 28 spines (Fig. 14E).

##### Size.

*Atyaephyra tuerkayi* is a large sized species with maximum carapace length to be 7.1 mm for ♀♀

##### Molecular characters.

A haplotype found in *Atyaephyra tuerkayi* sp. n. is not shared by any other species of *Atyaephyra*. Additionally, it differs from all the other species in the following nucleotide positions in the COI gene of *Atyaephyra desmarestii* specimen Dour1, position 174: guanine (G), position 207: adenine (A), position 246: adenine (A), position 318: thymine (T), position 321: adenine (A), position 339: adenine (A), position 357: cytosine (C), position 372: thymine (T), position 399: thymine (T), position 417: adenine (A) and position 441: cytosine (C). Finally, the mean genetic distances between *Atyaephyra tuerkayi* and the other species were ranging from 19.7% to 25.7% (Table 2).

##### Etymology.

*Atyaephyra tuerkayi* sp. n. is named after Professor Michael Türkay, in appreciation of his contribution to the study of Decapoda.

##### Distribution.

*Atyaephyra tuerkayi* sp. n. is found in the Nahr Al-Kabir River situated between Syria and Lebanon (see material examined and Fig. 1).

##### Remarks.

In addition to the type-material we investigated the morphology of the 23 female individuals (6 ovig.) and 7 males originating from Nahr Al-Kabir River (Fig. 1, stn 122; SMF 12189, SMF 12191, SMF 12192). All the individuals examined (including the sequenced ones) were morphologically identical. However, their placement to *Atyaephyra tuerkayi*, sp. n. has still to await sequencing. Since no male or ovigerous individual was sequenced observation regarding the form of the endopod of first male pleopod and number of eggs carried by the female were not included in the description. But observations were made in other individuals of the same sample and population and thus given here: endopod of first male pleopod expanded proximally and with a distal portion elongated and tapering, endopod with 9–16 spines arranged on a slightly curved inner margin and 9–11 setae arranged on outer margin. 430–450 eggs of 0.45–0.50 × 0.30–0.35 mm in size. Maximum carapace length to be 5.7 mm for ♂♂, 7.9 mm for ♀♀ and 7.6 mm for ovig. ♀♀.

Out of the 135 characters examined (see Appendix: Table 1) there were no morphological features distinguishing *Atyaephyra tuerkayi* sp. n. from *Atyaephyra desmarestii* and *Atyaephyra acheronensis* sp. n. However, *Atyaephyra tuerkayi* sp. n. can easily be distinguished from *Atyaephyra orientalis*, *Atyaephyra stankoi* and *Atyaephyra thyamisensis* by the presence of fewer mesial spines (Fig. 14H) (1–6) on terminal segment of third maxilliped (vs. 10–38 in *Atyaephyra orientalis*, *Atyaephyra stankoi* and *Atyaephyra thyamisensis*; Figs 4H, 6H, 8H) and by the basal endite of first maxilliped overreaching distal end of exopod (Fig. 14F) (vs. failing to reach or reaching distal end in *Atyaephyra orientalis*, *Atyaephyra stankoi* and *Atyaephyra thyamisensis*; Figs 4F, 6F, 8F). *Atyaephyra tuerkayi* sp. n. can be separated from *Atyaephyra strymonensis* by the presence of 1–3 post orbital rostral teeth (Fig. 13A) (vs. no post orbital teeth present leaving short unarmed proximal gap in *Atyaephyra strymonensis*; Fig. 9A) and by the endite of maxilla being 1.58–1.59 × as long as basal lower endite (Fig. 14G) (vs 1.77–1.95 in *Atyaephyra strymonensis*; Fig. 10G).

## Discussion

Given the highly structured nature of freshwater habitats and the limited potential for dispersal of the freshwater species (mainly due to natural barriers) in combination with the wide distribution of *Atyaephyra* in the Mediterranean region, a hypothesis under which several species are expected to be harbored in the genus seemed highly possible.

However, until recently, *Atyaephyra* was considered as a monotypic genus. Over the last 100 years many authors (Bouvier 1913, Holthuis 1961, Karaman 1972, Kinzelbach and Koster 1985, Al-Adhub 1987) have attempted to challenge this perception. However, the high intra- and inter-population variability, which made even the previously proposed subspecies questionable (Gorgin 1996, Anastasiadou et al. 2004) along with the lack of a complete series of samples covering all the known distribution of *Atyaephyra*, proved to be far more challenging than many taxonomists would ever anticipate.

In the latest revision of the *Atyaephyra* (Garcia Muñoz et al. 2009), which was based on the genetic information deriving from two mitochondrial genes (COI, 16S), two species were recognized while a third was proposed but without confirming it. In the current study seven species are defined, based both on morphological and molecular data. This difference in numbers is attributed to the limited geographical focus of the former study, which was primarily carried out on material collected from the Western Mediterranean area.

After an exhaustive study of a large number of specimens from 20 different countries and a thorough examination of more than 135 morphological characters, including somatometric distances, new characters were found which could differentiate species or groups of species within the *Atyaephyra*. One of these characters is the number of mesial spines on the terminal segment of the third maxilliped according to which two main groups can be distinguished. The first group is characterized by 10–38 mesial spines and comprises three species, *Atyaephyra thyamisensis* sp. n., *Atyaephyra stankoi*, *Atyaephyra orientalis* whereas the second by 1–8 mesial spines including the remaining four, namely *Atyaephyra desmarestii*, *Atyaephyra acheronensis* sp. n., *Atyaephyra strymonensis* sp. n.and *Atyaephyra tuerkayi* sp. n.

The species included in the first group can subsequently be distinguished by a series of features, e.g. presence-absence of a protruding pterygostomial angle, shape of antennular lobe and shape of endopod of first male pleopod. *Atyaephyra thyamisensis* sp. n., *Atyaephyra stankoi* and *Atyaephyra orientalis* are morphologically and phylogenetically well defined. In the phylogenetic tree they represent three well supported clades (16.7%–22.6% divergent from each other). In the second group, *Atyaephyra strymonensis* sp. n. is also a well defined species morphologically and can be distinguished from the remaining members by a combination of characters such as the lack of post orbital teeth, presence of a short unarmed proximal gap on rostrum and ratio of basal lower endite of maxilla in relation to the whole maxilla endite. The genetic divergence observed between *Atyaephyra strymonensis* sp. n. and its closest congeners by morphology is quite high (21.9%–25.4%). Thus, both morphological and molecular data show congruent patterns and jointly support its recognition as a distinct species within the genus. In addition, although *Atyaephyra strymonensis* sp. n. seems to be morphologically closer to the members of the second group e.g. *Atyaephyra desmarestii*, *Atyaephyra acheronensis* sp. n., *Atyaephyra tuerkayi* sp. n., genetically it is more closely related to the other two species of the first group from Greece (e.g. *Atyaephyra thyamisensis* sp. n.and *Atyaephyra stankoi*)with which it forms a strongly supported phylogroup (genetic divergence range: 11.9%–18.2%).

No diagnostic morphological characters were found to distinguish the species *Atyaephyra desmarestii*, *Atyaephyra acheronensis* sp. n. and *Atyaephyra tuerkayi* sp. n. from each other, a fact which is mainly caused by the high morphological variability observed in *Atyaephyra desmarestii*. However, their genetic distinctiveness coupled with their discrete geographical distribution provides enough evidence to distinguish the three species as distinct taxa.

The range of genetic divergence observed between the specimens of *Atyaephyra desmarestii* and of *Atyaephyra acheronensis* sp. n. (TrN distances: 5.9%–11.6%, Uncorrected p-distances: 5.3%–8.7%) is comparable to those found for other cryptic or sibling species of freshwater shrimps (e.g. Page et al. 2005a, Uncorrected p-distances: *Caridina* sp. A vs *Caridina* sp. B or C: 8.4–10.9%; *Caridina* sp. B vs *Caridina* sp. C: 6.7–8.8%), freshwater crabs (e.g. Jesse et al. 2011, Uncorected p-distances: interspecific variability between 14 *Potamon* species range: 3.1%–11.2%) as well as for other decapod sibling or well defined species (e.g. Jones and Macpherson 2007, TrN distances: interspecific variability between 14 *Munidopsis* species range: 1.5%–19.6%). The mean genetic divergence observed between *Atyaephyra desmarestii* and *Atyaephyra acheronensis* (8.3 %) was the smallest among the *Atyaephyra* species (remaining genetic distances ranging from 11.9 to 25.7%). This level of divergence was also evident in morphology, indicating a more recent speciation event within the genus (compared to the ones that gave rise to the other species of *Atyaephyra*) and thus less time for these two species to diverge both morphologically and genetically.

Furthermore, the fact that no haplotypes were shared between *Atyaephyra desmarestii* and *Atyaephyra acheronensis* sp. n. would suggest that the populations of shrimps from both species, although recently evolved, had independent evolutionary histories for a relatively long period of time. Additional support, although further research is still needed, comes from their geographical distribution since *Atyaephyra desmarestii* and *Atyaephyra acheronensis* sp. n. seems to be allopatric. *Atyaephyra acheronensis* is found in the western Balkan Peninsula, ranging from Croatia to Greece. In Greece, this species is found only on the west side of the mainland reaching most probably as far as South Peloponnese although with a remarkable fragmented distribution. In comparison *Atyaephyra desmarestii* is distributed in West-central Europe and North Africa. It should be noted here that the native distribution of *Atyaephyra desmarestii* is limited to Southern Europe and its presence in North-Central Europe up to the Danube River is believed to have been caused by its dispersal through the canals that were opened to connect the main rivers of Europe (Dhur and Massard 1995, Moog et al. 1999, Grabowski et al. 2005, Straka and Špaček 2009). Geographical barriers like the Alps and the Balkan mountains that isolated the Balkan drainages preventing faunal exchanges with the rest of Europe (Economou et al. 2007) could also account for this secluded population. Although, the current evidence deriving from mitochondrial data along with the geographic distribution supports the discrimination of *Atyaephyra acheronensis* as a distinct species, further support could come from additional mitochondrial sequence data (especially from the Balkan peninsula) as well as by combining information provided by nuclear sequence data.

The monophyly of the species *Atyaephyra desmarestii*, although supported by NJ, was poorly or not supported at all by BI and ML analyses, respectively. In the study of Garcia Muñoz et al. (2009) the monophyly of this species, based on the COI sequences, was strongly supported. This difference should be attributed to the larger number of sequences used in this study. *Atyaephyra desmarestii* (Millet, 1831) does not comprise a strongly supported genetically distinct group and appears as a not well resolved part of the phylogeny. However, the genetic distances observed within this group are quite small in comparison with the other *Atyaephyra* species and this in combination with the morphological data supports the consideration of all the populations inside this group as one taxonomic entity. More sequence and morphological data, especially from the area of South Portugal and Morocco (the monophyly of the species is strongly supported once the sequences originating from Morocco and South Portugal material are removed), as well as other molecular markers are needed in order for the relationships within *Atyaephyra desmarestii* to be clarified.

In the southwestern part of the Mediterranean area, only two species of *Atyaephyra* have been described to date: *Atyaephyra desmarestii* and *Atyaephyra rosiana*. These two species had been considered synonyms until Anastasiadou et al. (2008) resurrected *Atyaephyra rosiana* after studying material from São Barnabé River (Odelouca River) in South Portugal. In their study Garcia Muñoz et al. (2009), stated that the hypothesis of the two distinct species could not be supported although they did note some genetic variability in the specimens originating from South Iberian Peninsula. Similar results are obtained in the current study. Sequences from North African and South Iberian individuals presented a noticeable mean genetic divergence (3.1% and 2.3% respectively) from the rest of west European and Tunisian sequences, but although noticeable is still weak to support the hypothesis of different species. A high variability in morphological characters, especially in the individuals from the South Iberia was also observed. Characters such as the length and height of the rostrum (the tendency is for rostra to be longer and narrower) and the number of rows in maxilla basal lower endite (usually 15–18) varied greatly from the typical form present in North Iberia and the rest of Europe as well as Tunisia (shorter and broader rostra, 17–21 rows on maxilla basal lower endite). Genetic diversity among the South and North-central Iberia populations is observed in many other freshwater species whereas only in a few of them is it robust enough to justify distinct species (Doadrio and Carmona 2003, Durand et al. 2003, Sanjur et al. 2003). An explanation for this should be sought in the eventful geological history as many basins of the Iberian Peninsula almost dried up and the southwestern part of the Peninsula became completely isolated during the Messinian period (Sanjur et al. 2003). In addition, the genetic diversity observed mainly between the Moroccan and remaining populations should be sought again to the geological history and the isolation of the North-west Africa from Europe and where dispersal between these land mass, across the Gibraltar strait ceased to be an option since Pliocene (Sanjur et al. 2003).

The Tunisian populations, on the other hand, are more closely related to the western European ones, probably due to the past connections through the Sicily Strait with European populations (Butler et al. 1999).

The second cryptic species *Atyaephyra tuerkayi* sp. n. has been found only in the River Nahr Al-Kabir which is located along the borders of Lebanon with Syria. *Atyaephyra tuerkayi* sp. n. is completely isolated geographically from the other two morphologically closest to it species, *Atyaephyra desmarestii* and *Atyaephyra acheronensis* sp. n. In fact *Atyaephyra tuerkayi* sp. n. is surrounded by *Atyaephyra orientalis* populations which show a wide distribution from Turkey to Iraq. *Atyaephyra tuerkayi* sp. n. is genetically well discriminated from *Atyaephyra desmarestii* and *Atyaephyra acheronensis* (genetic distances are 23.0% and 22.2% respectively) as well as from *Atyaephyra orientalis* that is found in the adjacent areas (genetic distance is 19.7%). The genetic distances are among the highest observed between *Atyaephyra* species and by far exceed currently published records of intra-population variability of other fresh water decapods (e.g. Jesse et al. 2011). Furthermore, they are comparable with genetic distances of COI sequences described elsewhere for taxa recognized at the generic level (Avise 2000, Lefébure et al. 2006, Matzen da Silva et al. 2011). Therefore such an extensive differentiation should be attributed to speciation.

In the area of the Middle East, two subspecies were previously described, *Atyaephyra desmarestii orientalis* Bouvier, 1913 and *Atyaephyra desmarestii mesopotamica* Al-Adhub, 1987. However, no observable morphological characters where found that could differentiate them (see remarks of *Atyaephyra orientalis*). Furthermore, although the genetic distances within the *Atyaephyra orientalis* phylogroup were high (0.9%–10.2%) no firm conclusion could be drawn whether the hypothesis of multiple species is valid or not. Sequences from Orontes River (topotypical location of *Atyaephyra desmarestii orientalis*) and from Shatt Al-Arab River (topotypical location of *Atyaephyra desmarestii mesopotamica*) presented a noticeable mean genetic divergence (5.0%) but still not strong enough to support the hypothesis of different species. Detailed future studies on the morphological and genetic variability within the samples of *Atyaephyra* distributed throughout the Middle East will help clarify the relationships between the populations in this region, however given the present data, only one species is considered to exist, *Atyaephyra orientalis*.

Four species (*Atyaephyra acheronensis* sp. n., *Atyaephyra thyamisensis* sp. n., *Atyaephyra stankoi*, and *Atyaephyra strymonensis* sp. n.) were found to co-exist in Greece with well defined and clearly separated distributions. Only two species (*Atyaephyra acheronensis* sp. n. and *Atyaephyra thyamisensis* sp. n.) were found to co-exist in the same river (River Louros, Epirus). Multiple individuals collected from the Louros estuary and further upstream, dating back to 1977 until 2001 were examined. These specimens were all identified as *Atyaephyra thyamisensis* sp. n. However, in a recent sample (2012) both species were found. Probably, this could be attributed to fish transfers or translocation where shrimps could have accidentally been introduced. Additionally, the distance between the estuaries of the Rivers Louros and Acherontas is less than 30 km making human mediated dispersal, between the two watersheds, highly possible. Furthermore, numerous translocations of fish were made within Greece over the last 70 years (Economidis et al. 2000) making this scenario even more justified. However, the natural co-existence of the two species cannot be entirely excluded.

It is surprising that four out of the seven *Atyaephyra* species examined for the present study are recorded from Greece and three of these are endemic. Greece is considered to be a faunal and floral biodiversity hot spot within the Mediterranean region where freshwater fauna is not an exception (Reyjol et al. 2007, Jesse et al. 2011). Jesse et al. (2011) after studying the diversity of the freshwater *Potamon* crabs, revealed the existence of 14 species within the greater Mediterranean region. Eight of these species (three endemic and five with limited distribution in adjacent countries) were found in Greece. High diversity and endemism is recorded in other freshwater groups too, such as fishes. Greece harbours the largest number of fish species of any region in the Mediterranean basin where the number of endemic species exceeds 45% of the total number of native species (130) recorded (Economou et al. 2007, Blondel et al. 2010). Freshwater endemism in Greece is considered as one of the highest in the Mediterranean region and has been ascribed to its eventful geological history combined with complex climatic events (Bobori et al. 2001, Economou et al. 2007).

The importance of morphology versusmolecular data in order to resolve the phylogeny of a taxon still provides a forum for scientific debate (Tautz et al. 2003, Blaxter 2004, Page et al. 2005b). Although additional work is needed towards the exhibited morphological variability within the genus, the data provided by the present study demonstrate a case in which conventional and molecular taxonomy do not provide different patterns but, rather, complimentary. Finally, an additional step was taken by considering the molecular validation of the two cryptic species which couldn’t be supported by morphological data alone. It seems, therefore, that when both molecular and morphological effort is combined towards a “total evidence” approach a whole greater than the sum of its parts emerges which is instrumental in our understanding the diversity of life (Page et al. 2005b).

## Supplementary Material

XML Treatment for Atyaephyra

XML Treatment for Atyaephyra desmarestii

XML Treatment for Atyaephyra orientalis

XML Treatment for Atyaephyra stankoi

XML Treatment for Atyaephyra thyamisensis

XML Treatment for Atyaephyra strymonensis

XML Treatment for Atyaephyra acheronensis

XML Treatment for Atyaephyra tuerkayi

## Appendix

List of morphological characters studied and photos of *Atyaephyra desmarestii* and "*Atyaephyra rosiana*" material examined by Bouvier (1913). (doi: 10.3897/zookeys.229.3919.app) File format: Mictosoft Office Document (docx).

**Explanation note:** A list of 135 morphological characters (67 meristic and 68 somatometric distances) examined in *Atyaephyra* species is given. Schematic drawings of *Atyaephyra* appendages showing the studied somatometric characters are supplied. Furthermore, photos of *Atyaephyra desmarestii* and *Atyaephyra rosiana* material, collected from Maine et Loire (France) and Coimbra (Portugal) respectively, examined by Bouvier (1913) are given.

**Copyright notice:** This dataset is made available under the Open Database License (http://opendatacommons.org/licenses/odbl/1.0/ ). The Open Database License (ODbL) is a license agreement intended to allow users to freely share, modify, and use this Dataset while maintaining this same freedom for others, provided that the original source and author(s) are credited.
